# Understanding SERS Spectral Shape Variability through Substrate Optics, Molecular Orientation, and Unsupervised Clustering

**DOI:** 10.1021/acs.jpcc.5c08676

**Published:** 2026-03-24

**Authors:** Amit Kumar, Fengbo Ma, Xianyan Chen, Yiping Zhao

**Affiliations:** † Department of Physics and Astronomy, The University of Georgia, Athens, Georgia 30602, United States; ‡ School of Electrical and Computer Engineering, College of Engineering, The University of Georgia, Athens, Georgia 30602, United States; § Department of Epidemiology & Biostatistics, College of Public Health, The University of Georgia, Athens, Georgia 30602, United States

## Abstract

Surface-enhanced Raman scattering (SERS) enables ultrasensitive molecular detection, yet systematic variations in spectral shape (not just signal intensity) often limit reproducibility and interpretation. Here, we present a combined experimental and chemometric study that elucidates how substrate optical response and molecular adsorption orientation jointly govern SERS spectral variability. Using 1,2-bis­(4-pyridyl) ethylene (BPE) on oblique-angle-deposited silver nanorod (AgNR) substrates as a model system, we constructed a comprehensive spectral data set spanning six controlled experimental conditions, including defect mapping, batch-to-batch variation, nanorod length tuning, concentration-dependent drop-casting, static immersion, and real-time immersion measurements. Hierarchical cluster analysis (HCA) partitions the spectra into seven reproducible clusters with distinct average spectral shapes, separating low- and high-signal-to-noise regimes and revealing systematic evolution of relative intensities among the five characteristic BPE modes. By correlating cluster membership with experimental metadata, we show that specific spectral shapes are strongly associated with defined physical conditions, including surface defects, nanorod geometry, analyte concentration, and adsorption dynamics. To interpret the cluster-dependent spectral shapes, we introduce intensity web plots under three normalization strategies that isolate different governing physics: absolute intensities emphasize overall electromagnetic enhancement and analyte coverage; normalization by the sum of the five peak intensities suppresses global scaling and highlights substrate-dependent optical reweighting of Raman bands; and normalization to the 1015 cm^–1^ mode provides an internal reference that accentuates orientation-selective enhancement. Together, these results establish a physics-informed, data-driven framework for better understanding the origins of SERS spectral shape changes under complex experimental conditions.

## Graphical abstract

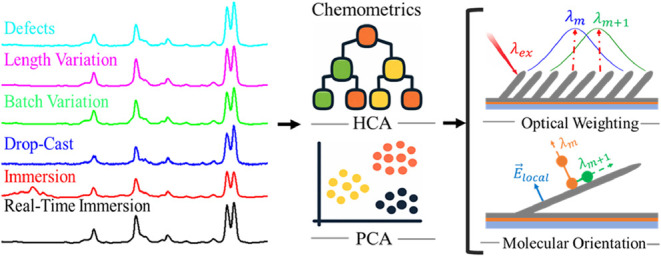

## Introduction

Surface-enhanced Raman spectroscopy (SERS) enables ultrasensitive, label-free molecular detection by amplifying Raman scattering through localized electric field enhancement and charge-transfer interactions at metal surfaces.^1^ These enhancement mechanisms provide exceptional chemical specificity and sensitivity, making SERS attractive for applications ranging from biomedical diagnostics and environmental monitoring to food safety and chemical threat detection.^2^ Advances in nanofabrication and measurement protocols have further improved reproducibility, positioning SERS as a promising platform for practical sensing applications. Despite its remarkable sensitivity and versatility, the interpretation of SERS spectra extends beyond simple identification of vibrational fingerprints. In many cases, the observed spectrum is not solely determined by the intrinsic vibrational modes of the analyte. Instead, the spectral shape is strongly modulated by two intertwined factors: the optical response of the plasmonic substrate and the orientation of molecules adsorbed at the surface.^3,4^ Substrate morphology controls the spatial distribution and spectral weighting of localized electromagnetic fields, while adsorption geometry governs how individual vibrational modes couple to the excitation and localized electromagnetic fields. As a result, relative peak intensities may vary, certain bands may be selectively enhanced or suppressed, and reproducible spectra may emerge only under specific experimental conditions.

The influence of substrate optical properties on SERS spectra has been extensively documented. Variations in nanostructure morphology, arrangement, and size reshape the localized surface plasmon resonance (LSPR) and redistribute SERS hotspots, leading to wavelength-dependent reweighting of Raman modes.^5–7^ Numerous studies have shown that tuning nanorod aspect ratio, length, and arrangement can selectively enhance specific vibrational modes by modifying resonance overlap with excitation and scattered wavelengths. For example, Yamamoto et al. showed that morphological changes shift the LSPR and alter peak ratios for the same molecule.^3^ Orendorff et al. reported that nanorod aspect ratio governs resonance matching and enhancement efficiency,^8^ while Lin et al.^9^ and Huang et al.^10^ found subtle but systematic peak-ratio changes even under nominally fixed resonance conditions. Polarization-dependent enhancement in tilted Ag nanorod arrays,^11^ deposition-parameter effects,^12^ and recent theoretical treatments of wavelength-dependent enhancement and attenuation^13^ further establish substrate optics as a major contributor to SERS spectral variability.

At the same time, molecular orientation at the plasmonic surface can independently and strongly influence the spectral shape. According to the surface selection rule, enhancement efficiency depends on the alignment between a vibrational mode’s Raman polarizability tensor and the local electric field.^14^ Vibrations aligned with the surface normal are preferentially enhanced, while tangential modes may be weak or absent.^15,16^ Field gradients can further activate or suppress modes depending on adsorption geometry.^17^ Experimental studies have shown that adsorption configuration alters peak ratios for amino acids, peptides, DNA, and small organic molecules,^18–21^ while chemisorption geometry can induce both intensity redistribution and frequency shifts.^22,23^ Orientation effects are also dynamic: molecular reorientation during adsorption can produce time-dependent spectral evolution,^24^ and orientation averaging depends on molecular size and packing density.^21^ These results demonstrate that spectral shape variations cannot be attributed to substrate effects alone.

In practice, substrate optical properties and molecular orientation often act simultaneously, thereby complicating interpretation. Wu et al. showed that nanoparticle morphology influences both plasmon modes and adsorption behavior,^25^ highlighting the coupling between electromagnetic and chemical factors. While many prior studies intentionally engineer substrates or binding geometries to isolate one effect, most practical SERS substrates, particularly those with random or network-like morphologies, exhibit relatively subtle optical variations. Moreover, common probe molecules such as 1,2-bis­(4-pyridyl) ethylene (BPE) or rhodamine 6G lack a unique preferred adsorption orientation. Nevertheless, clear and reproducible spectral shape variations are frequently observed, indicating that even modest changes in enhancement conditions or adsorption environment can systematically reshape SERS spectra. A general framework for disentangling or understanding the roles of these effects under realistic experimental conditions remains lacking.

More importantly, the absence of such a framework carries practical consequences. In complex SERS measurements, multiple physical and chemical factors, including electromagnetic spectral reweighting, adsorption geometry, surface heterogeneity, and analyte loading, can act concurrently. Without explicitly separating these contributions, spectral variations risk being interpreted phenomenologically, potentially leading to misattribution of physical effects or overinterpretation of chemical changes. Establishing a physics-grounded baseline that distinguishes electromagnetic enhancement from orientation-dependent Raman tensor projection is therefore a necessary step toward reliable interpretation in increasingly complex sensing environments.

In this work, we combine systematic experimental design with chemometric analysis to elucidate the origins of SERS spectral shape variations for BPE adsorbed on geometrically random silver nanorod (AgNR) substrates. Six complementary experimental conditions are employed to independently probe substrate-related factors: defect mapping, batch-to-batch variation, nanorod length tuning and adsorption-related factors, including concentration-controlled drop-casting, static immersion, and real-time immersion. Hierarchical cluster analysis (HCA) objectively partitions the spectra into low- and high-signal-to-noise (SNR) regimes with reproducible spectral subtypes, while principal component analysis (PCA) confirms that cluster separation is dominated by systematic redistribution among the five characteristic BPE vibrational modes rather than noise or baseline artifacts. The results indicate that substrate-driven electromagnetic enhancement and orientation-dependent vibrational coupling play distinct but complementary roles in shaping SERS spectra, governing signal quality and relative peak intensities, respectively. This physics-informed, data-driven framework provides a clear basis for understanding SERS spectral shape variations on realistic substrates.

## Experimental Methods

### Materials

1,2-Bis­(4-pyridyl) ethylene (BPE) (Sigma-Aldrich, 98%) was obtained as a model analyte. Sulfuric acid (Fisher Scientific, 98%), ammonium hydroxide (Fisher Scientific, 98%), and hydrogen peroxide (Fisher Scientific, 30%) were used to clean glass slides (Gold Seal, Part# 3010). Silver (Kurt J. Lesker, 99.999%) and titanium pellets (Kurt J. Lesker, 99.995%) were purchased as the evaporation materials. Pure water (Sigma-Aldrich) and methyl alcohol (Sigma-Aldrich) were used as solvents throughout the experiments.

### Silver Nanorod Substrate Fabrication

AgNR substrates were fabricated using the oblique angle deposition (OAD) technique via electron beam evaporation.^26,27^ Prior to deposition, glass substrates were thoroughly cleaned using a piranha solution (4:1 mixture of concentrated sulfuric acid and hydrogen peroxide) for 10 min to remove organic contaminants and improve surface wettability. After cleaning, the slides were rinsed with copious amounts of deionized water and dried under a nitrogen stream. A base layer of titanium (10 nm) and a silver film (100 nm) were first evaporated onto the glass slides at normal incidence to the substrate surface at rates of approximately 2 Å/s and 3 Å/s, respectively. The substrates were then rotated to an angle of ∼86° with respect to the vapor flux direction. AgNR substrates were grown at this oblique angle with a nominal deposition rate of 3 Å/s and a deposition pressure of 1 × 10^–6^ Torr. A schematic illustration of the AgNR structure is provided in Figure S1a of the Supporting Information (SI), and a representative electron micrograph of the substrate is shown in Figure S1b. The substrate consists of randomly distributed, elongated, and tilted nanorods. Individual nanorods exhibit irregular morphology, with diameters that vary along their length and occasional branching or inter-rod connections. All AgNR substrates were argon-plasma cleaned in a custom-built inductively coupled RF plasma chamber before applying the BPE solution.

### SERS Sample Preparations

BPE was selected as the model analyte because of its well-characterized vibrational modes and strong affinity for silver via pyridyl nitrogen coordination.^28,29^ The presence of two pyridyl groups allows BPE to adopt multiple adsorption geometries (upright, tilted, or flat) on the Ag surface. These distinct orientations are known to produce measurable changes in relative Raman peak intensities. Leveraging these properties, we designed six complementary experimental configurations to systematically examine how substrate characteristics and adsorption behavior jointly affect SERS spectra. These configurations target local morphology, fabrication reproducibility, nanorod geometry, adsorption conditions, and adsorption kinetics. A detailed description of each factor is provided below.

(a) **Defect mapping:** During OAD, surface defects occasionally formed due to dust particles, organic residues, or surface irregularities that disrupted the self-shadowing growth process. These regions contained broken, misaligned, or missing nanorods. To assess the impact of such defects, spectra were collected from three regions: directly on defects, near defect edges, and uniform off-defect areas (Figure 1a). A 10^–5^ M BPE solution in methanol was drop-cast onto plasma-cleaned AgNR substrates and allowed to dry. Defects were identified optically, and a mapping grid (1000 μm × 920 μm, 100 μm step size) was defined around each defect, yielding 92 spectra.
(b) **Batch-to-batch variation:** To evaluate fabrication reproducibility, ten batches of AgNR substrates produced over a three-year period were compared. Although all substrates were fabricated using identical OAD parameters, minor variations in chamber conditions, silver source state, and handling procedures could introduce subtle differences. A 10^–5^ M BPE solution was drop-cast onto each plasma-cleaned substrate, and nine spectra were collected from spatially distinct but visually uniform regions, yielding 90 spectra in total.
(c) **Nanorod length variation:** To isolate the effect of nanorod length, AgNR substrates with lengths of 200, 400, 600, 800, and 1000 nm were fabricated in a single deposition run using a custom shutter-controlled mounting assembly. This approach ensured identical deposition conditions while varying only nanorod length. A 10^–5^ M BPE solution was drop-cast onto each substrate, and spectra were collected from multiple locations.
(d) **Drop-cast adsorption:** To investigate concentration-dependent spectral changes under static adsorption conditions, a dilution series of BPE (10^–3^–10^–12^ M) was prepared in methanol. Ten AgNR substrates from the same batch were used to ensure consistent plasmonic properties. For each concentration, 5 μL of solution was drop-cast onto a plasma-cleaned substrate and allowed to dry. Subsequently, nine spectra were collected per substrate, yielding 90 spectra in total (Figure 1b).
(e) **Static immersion adsorption:** To compare equilibrium adsorption with drop-casting, immersion-based preparation was employed using the same BPE concentration range (10^–3^–10^–12^ M). For each concentration, a clean AgNR substrate was immersed in the solution for 30 min, then removed, air-dried, and measured at multiple locations. A total of 90 spectra were collected (Figure 1c).
(f) **Real-time immersion:** To directly monitor adsorption kinetics and time-dependent spectral evolution, real-time immersion experiments were conducted. A PDMS well (4 mm in diameter) was mounted on AgNR substrates, filled with 15 μL of BPE solution (10^–5^ M), and sealed with a glass coverslip to prevent evaporation (Figure 1d). SERS spectra were collected at 1 min intervals from initial adsorption to steady state.

**Figure 1:**
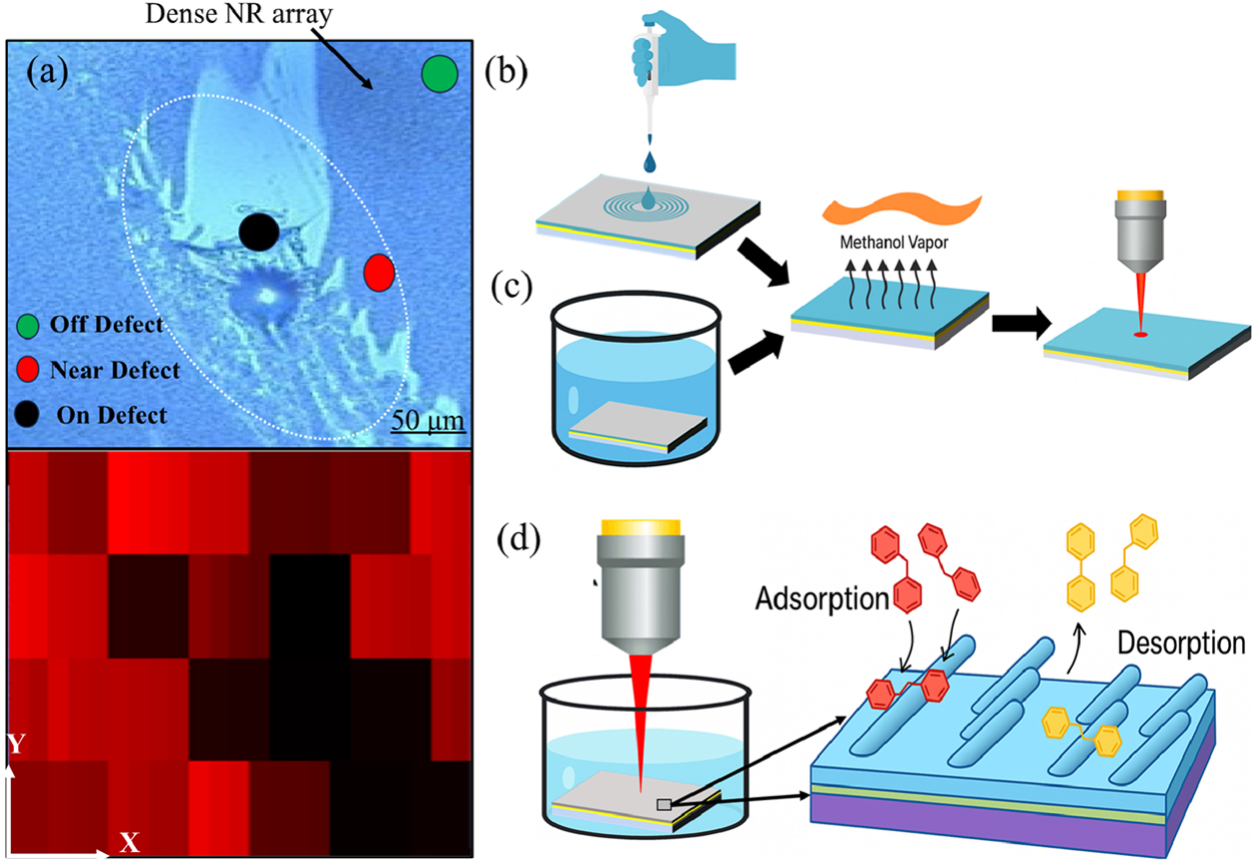
Experimental conditions for BPE SERS analysis: (a) Defect mapping showing measurements on, near, and off a substrate defect; (b) Drop-cast adsorption with drying before measurement; (c) Static immersion adsorption with postdrying measurement; (d) Real-time immersion measurements for monitoring adsorption–desorption dynamics.

### SERS Measurement Conditions

All SERS measurements were performed using an InVia Renishaw Raman spectrometer equipped with a 785 nm diode laser. The laser was focused onto the sample surface using a 5× microscope objective, which offered a balance between spot size and signal collection efficiency. The laser power at the sample was set to 7 mW. Spectra were collected with an integration time of 10 s and one accumulation per measurement.

### Spectral Data Preprocessing

Raw spectral data were initially acquired using Renishaw WiRE software, where baseline correction was performed with the built-in algorithm. All subsequent preprocessing steps were carried out using SpectraGuru (https://spectraguru.org/). These steps included: (a) despiking to remove cosmic ray artifacts; (b) normalization by the mean intensity to standardize signal scales across measurements; (c) wavenumber interpolation to ensure consistent spectral resolution and alignment; (d) labeling based on the experimental condition; and (e) assembly into a unified CSV format suitable for chemometric input.

### Hierarchical Cluster Analysis (HCA)

HCA is an unsupervised statistical method used to identify natural groupings within multivariate data sets based on spectral similarity. In this study, HCA was applied to determine whether an intrinsic structure exists within the BPE SERS data set and to assess how experimental factors influence spectral shape. By organizing spectra according to similarity rather than prior labels, HCA enables the objective identification of distinct spectral groups and facilitates interpretation of the physical origins of spectral variability.^30^ A key step in HCA is defining a similarity metric between individual spectra. Here, we used the Euclidean distance, which provides a direct measure of overall spectral differences. Pairwise spectral similarity was quantified using the Euclidean distance computed on the intensity vectors across all Raman shift variables. The pairwise Euclidean distance *d*_*ij*_ between spectra *i* and *j* was calculated as,

1 $$d_{\mathrm{ij}}\left({\Delta v}^{\left(m\right)}\right)=\sqrt{\sum_{m=1}^{R}{\left(I_{i}\left({\Delta v}^{\left(m\right)}\right)-I_{j}\left({\Delta v}^{\left(m\right)}\right)\right)}^{2}}$$

Where *R* is the total number of Raman-shift data points (wavenumbers), Δ*v* ^(*m*)^ denotes the *m-*th Raman shift, and *I*_*i*_(Δ*v* ^(*m*)^) and *I*_*j*_(Δ*v* ^(*m*)^) are the SERS intensities of spectra *i* and *j*, respectively, at Δ*v* ^(*m*)^. Clustering was performed using Ward’s linkage method,^31^ which merges clusters by minimizing the increase in total within-cluster variance at each step. This criterion is based on the squared Euclidean distance between cluster centroids and is given by

2 $$D\left(A,B\right)=\frac{N_{A}N_{B}}{N_{A}+N_{B}}{∥{\mathrm{I̅}}_{A}-{\mathrm{I̅}}_{B}∥}^{2}$$

where *N*_A_ and *N*_B_ are the number of spectra in clusters A and B, respectively, and ${\mathrm{I̅}}_{A}=\frac{1}{N_{A}}\sum_{i\in A}I_{i}$ and ${\mathrm{I̅}}_{B}=\frac{1}{N_{B}}\sum_{i\in B}I_{i}$ are the corresponding cluster centroids (mean spectral vectors). Each spectrum is represented as ***I***_*i*_ = [*I*_*i*_(Δ*v* ^(1)^), *I*_*i*_(Δ*v* ^(2)^)···*I*_*i*_(Δ*v* ^(*R*)^)]. Ward’s method favors compact, homogeneous clusters and is therefore well suited for SERS spectral analysis.

The hierarchical clustering process yields a dendrogram, which visually represents the sequence of cluster mergers from individual spectra to a single unified cluster. The dendrogram was used to identify meaningful spectral groupings, including noise-dominated spectra, high-quality SERS signals, and subclusters associated with distinct experimental conditions. All analyses were performed in Python using the *scipy* package,^32^ and dendrograms were generated with *matplotlib*.^33^ The optimal cluster cut level was determined through a combination of dendrogram inspection and consistency with known experimental variations.

Following hierarchical clustering, the optimal number of natural clusters in the SERS data set was determined using two complementary quantitative criteria: the Elbow method and the Silhouette score analysis. Together, these approaches balance cluster compactness, separation, and interpretability.

The Elbow method evaluates the total within-cluster sum of squares (inertia) as a function of the number of clusters *K*.^34^ Inertia quantifies cluster compactness by summing the squared Euclidean distances between each SERS spectrum and the centroid of its assigned cluster

3 $$\mathrm{Inertia}\left(K\right)=\sum_{i=1}^{N}{∥I_{i}-{\mathrm{I̅}}_{c\left(i\right)}∥}^{2}$$

where *N* is the total number of spectra, ***I* ®**_*c*(*i*)_ is the centroid of the cluster to which the *i* spectrum belongs, and *c*(*i*) denotes the cluster assignment of spectrum *i*. As *K* increases, inertia decreases monotonically. The optimal cluster number is identified at the “elbow” point, beyond which further increases in *K* yield only marginal reductions in inertia, indicating diminishing returns in cluster compactness.

The Silhouette method complements the Elbow analysis by assessing the quality of cluster separation.^35^ For each spectrum *i*, the silhouette score *S*(*i*) is calculated as

4 $$S\left(i\right)=\frac{b\left(i\right)-a\left(i\right)}{\max\left\{a\left(i\right),b\left(i\right)\right\}}$$

where *a*(*i*) is the average Euclidean distance between spectrum *i* and all other spectra within the same cluster, and *b*(*i*) is the minimum average distance between spectrum *i* and spectra in any other cluster. *S*(*i*) ranges from −1 to +1, with values close to +1 indicating that spectra are well clustered, values near 0 suggesting overlapping clusters, and negative values reflecting possible misclassification. The average silhouette score across all spectra is used as an overall measure of clustering quality for each candidate cluster number *K*, with the optimal number of clusters corresponding to the maximum average silhouette score.

### Principal Component Analysis (PCA)

To further interrogate the variance structure and validate the physical relevance of the HCA-derived clusters, PCA^36^ was performed on the clustered spectra. PCA projects high-dimensional spectral data onto a set of orthogonal components that capture the dominant sources of variance. For a data set represented by an *N* × *p* data matrix *X*, the covariance matrix *C* is given by

5 $$C=\frac{1}{N-1}X^{T}X$$

where *N* is the number of spectra and *p* is the number of Raman shift (wavenumber) variables.

PCA seeks the eigenvectors *w*_*r*_ and corresponding eigenvalues λ_*r*_ of the covariance matrix according to

6 $$Cw_{r}=\lambda_{r}w_{r}$$

where each eigenvector *w*_*r*_ defines the *r*-th principal component direction in spectral space, and the associated eigenvalue λ_*r*_ quantifies the variance explained by that component. Projection of the spectra onto these principal component directions yields PCA scores, while the component loadings provide insight into the vibrational modes contributing most strongly to spectral variance. PCA score distributions and loading profiles were therefore used to interpret cluster separation in terms of systematic variations in vibrational-mode intensities.

### Metadata Labeling for Statistical Analysis

Following unsupervised hierarchical cluster analysis, experimental metadata were refined, and raw experimental labels were consolidated into physically meaningful categories. Defect-mapping spectra were grouped as defect-on (acquired on or immediately surrounding visible defect regions as shown by the dashed circle in Figure S6) and defect-off (acquired from structurally uniform regions away from defects). Nanorod-length-dependent measurements were grouped as short length (200 nm) and long length (400–1000 nm). Drop-cast adsorption spectra were grouped by BPE concentration into high (10^–3^–10^–5^ M), medium (10^–6^–10^–8^ M), and low (10^–9^–10^–12^ M) ranges, while immersion-based experiments used the same high and medium ranges with the low group defined as 10^–9^–10^–10^ M. Real-time immersion spectra were grouped into short-time (<5 min) and long-time (>5 min) regimes.

To relate unsupervised HCA results to experimental conditions, spectra were statistically summarized using two complementary metadata-based normalizations. A cluster-normalized (row-wise) representation was used, in which percentages within each HCA cluster sum to 100%. In addition, a condition-normalized (column-wise) representation was used, in which percentages within each experimental condition sum to 100%. These normalizations were applied solely for postclustering statistical analysis and did not influence the unsupervised HCA or PCA.

## Results and Discussion

### Potential Mechanisms to Modify the Relative SERS Intensities

The relative SERS intensity changes observed in this work can be interpreted using two complementary physical mechanisms: substrate-dependent optical effect and molecular orientation effect. Together, these mechanisms provide the basis for understanding why certain Raman bands strengthen or weaken under nominally identical measurement conditions.

### Substrate-Dependent Optical Effect

The plasmonic response of a SERS substrate plays a central role in determining both the overall signal strength and the relative intensities of individual Raman bands. Even when the analyte and excitation conditions remain fixed, variations in nanorod morphology, porosity, or structural disorder can modify the plasmonic response of the substrate, altering the near-field enhancement experienced at different Raman wavelengths as well as the optical response of the SERS scattering pathway.

In general, the SERS intensity of a vibrational mode *m* depends on the product of the local field enhancement at the excitation wavelength λ_ex_ and at its Stokes-shifted scattered wavelength λ_sc,*m*_. The electromagnetic enhancement can be expressed as^3,37–39^

7 $$I_{m}\propto {\left|{\mathrm{E⃗}}_{\mathrm{loc}}\left(\lambda_{\mathrm{ex}}\right)\right|}^{2}{\left|{\mathrm{E⃗}}_{\mathrm{loc}}\left(\lambda_{\mathrm{sc},m}\right)\right|}^{2}{\left|{ê}_{\mathrm{sc}}\cdot {\vec{\mathrm{\alpha ⃗}}}^{\left(m\right)}\cdot {ê}_{\mathrm{local}}\right|}^{2}$$

where *E⃗*_loc_(λ) is the local electric field at the excitation (λ_ex_) or scattered (λ_sc,*m*_) wavelength, ${\vec{\mathrm{\alpha ⃗}}}^{\left(m\right)}\approx \left(\frac{\partial \vec{\mathrm{\alpha ⃗}}}{\partial Q_{v}}\right)$ is the Raman polarizability tensor of mode *m*, and ${ê}_{\mathrm{sc}},{ê}_{\mathrm{local}}=\frac{{\mathrm{E⃗}}_{\mathrm{local}}}{\left|{\mathrm{E⃗}}_{\mathrm{local}}\right|}$ are the scattered and local field directions. Equation 7 highlights that each Raman band is weighted by the substrate’s wavelength-dependent enhancement at both the excitation and scattered wavelengths. Such wavelength-dependent plasmonic modulation of Raman line shapes has been discussed extensively in the literature, including studies demonstrating excitation- and scattering-wavelength-dependent enhancement effects in complex plasmonic systems.^40–42^ In addition, in backscattering geometries, additional optical attenuation occurs as scattered light propagates through the porous AgNR film. Zhao et al. showed that this attenuation can be modeled by Fresnel’s equations and the absorbance of the SERS substrates can also play a dominant role.^13^ Therefore, small shifts in LSPR position or absorption strength can selectively amplify or suppress Raman bands depending on their scattered wavelengths.

Figure 2 presents a conceptual illustration of wavelength-dependent electromagnetic reweighting of Raman modes. We consider two Raman modes, λ_*m*_ and λ_*m*+1_, that scatter at slightly different wavelengths, and two SERS substrates exhibiting slightly different LSPR responses. When λ_*m*_ lies closer to the substrate LSPR wavelength λ_LSPR_ (Figure 2a), this mode experiences stronger enhancement than λ_*m*+1_, producing the spectral shape shown in Figure 2b according to eq 7. Conversely, when the LSPR shifts such that the scattering wavelength λ_*m*+1_ is closer to λ_LSPR_ (Figure 2c), the relative SERS intensities can invert (Figure 2d), with λ_*m*+1_ becoming dominant and λ_*m*_ suppressed. This example highlights the key prediction of the optical model: even with identical analyte and measurement conditions, small LSPR shifts in SERS substrates can dramatically alter the relative intensities of Raman bands through purely wavelength-dependent electromagnetic effects.

**Figure 2:**
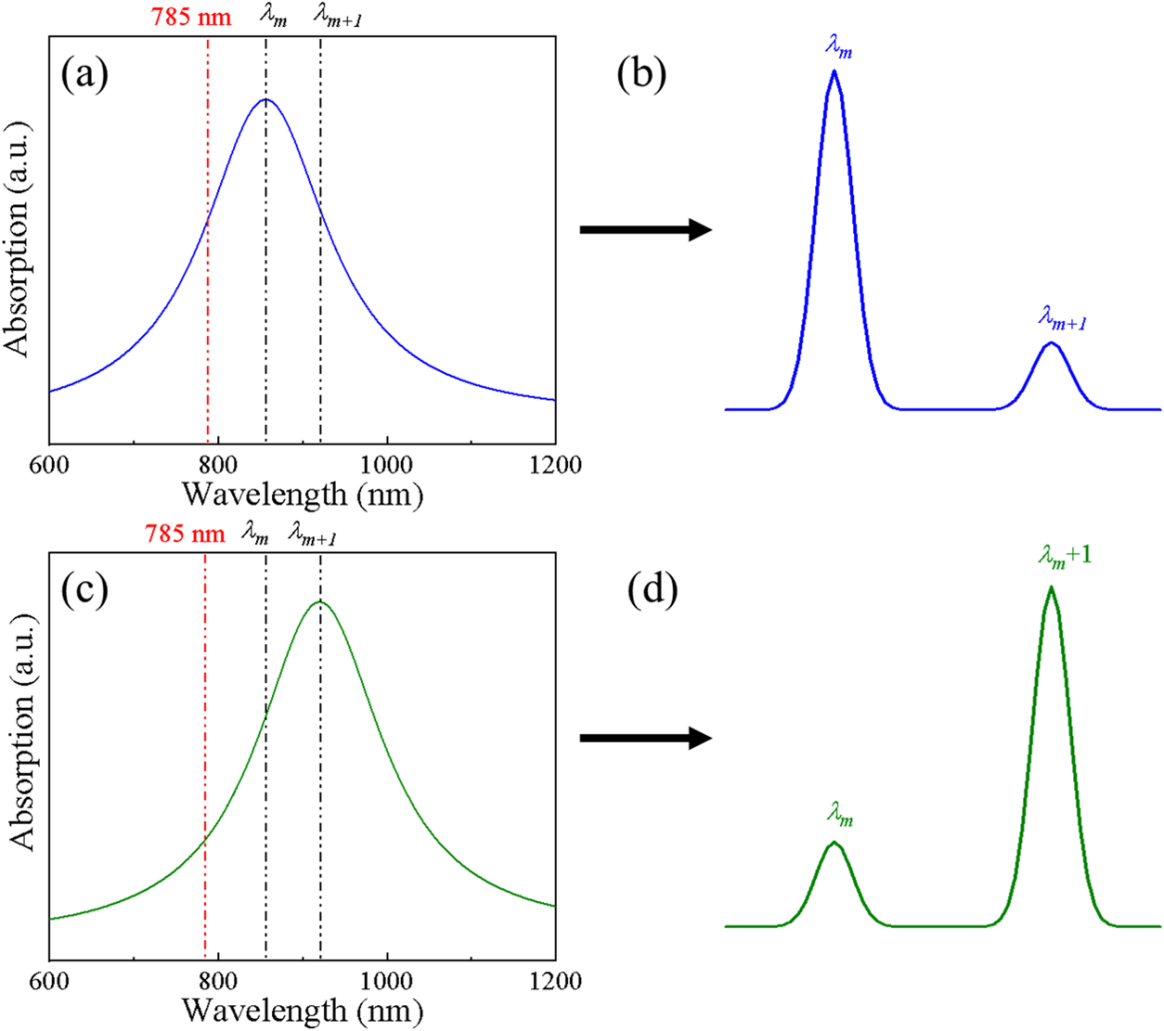
Schematic illustration of substrate-dependent optical reweighting of Raman mode intensities in SERS. (a) An *m*-th vibrational mode with scattering wavelength λ_*m*_ close to the substrate LSPR wavelength λ_LSPR_, producing the spectral shape shown in (b). (c) A different vibrational mode λ_*m*+1_ near λ_LSPR_, leading to the spectral shape shown in (d).

While the LSPR-based description of electromagnetic enhancement is conceptually straightforward for isolated plasmonic nanoparticles, the AgNR-on-Ag-film substrates investigated here form a coupled quasi-three-dimensional plasmonic network. The tilted nanorod arrays, together with the underlying Ag film, give rise to collective plasmonic coupling and a broadband optical response rather than a single, well-defined LSPR peak.^27^ To experimentally characterize this behavior, we measured the reflectance spectra for representative nanorod lengths and derived the corresponding effective extinction spectra (Figures S2–S3 of SI). The measured spectra exhibit broadband behavior in the 400–800 nm wavelength range, without the emergence of a sharp, length-dependent LSPR peak. At the 785 nm excitation wavelength used in SERS measurements, the reflectance varies between ∼69–81% across the tested nanorod lengths, corresponding to effective extinction values of ∼0.21– 0.36. These extinction values are comparable to those typically reported for discretized nanostructures exhibiting LSPR behavior.^43,44^

It should be noted that far-field extinction reflects an averaged optical response and does not directly represent the localized near fields in SERS hotspots. Although the AgNR substrates exhibit a broadband rather than a sharp LSPR peak, the near-field amplitude still varies with wavelength. Because SERS intensity scales as |*E⃗*_loc_(λ_ex_)|^2^|*E⃗*_loc_(λ_sc,*m*_)|^2^, even gradual spectral variations across a broadband response can differentially weight Raman modes that scatter at slightly different wavelengths. Thus, broadband plasmonic systems can induce mode-selective intensity redistribution analogous to that produced by discrete LSPR shifts.^45^ Small geometry-dependent modifications of the collective nanorod network can therefore selectively amplify or suppress specific vibrational bands through the same wavelength-dependent electromagnetic mechanism described in eq 7.

### The Molecular Orientation Effect

While the optical model describes how the substrate redistributes Raman intensities through wavelength-dependent electromagnetic enhancement, eq 7 shows that SERS intensity also depends critically on the Raman tensor projection term ${\left|{\hat{e}}_{\mathrm{sc}}\cdot {\vec{\mathrm{\alpha ⃗}}}^{\left(m\right)}\cdot {\hat{e}}_{\mathrm{local}}\right|}^{2}$ . This term governs how each vibrational mode couples to the local electric field and therefore provides the fundamental origin of orientation-dependent spectral variations. Figure 3 schematically illustrates the geometric framework used to analyze this tensor projection. The excitation laser is *p*-polarized and incident in the *y–z* plane. The Ag nanorod axis is tilted relative to the substrate normal, and the local surface-normal direction *n̂* is defined at the nanorod surface. The BPE molecule is adsorbed on the nanorod, with its molecular principal axes oriented relative to *n̂*. The arrows in Figure 3e represent the dominant polarizability-change directions (tensor anisotropy) of the five characteristic BPE vibrational modes, rather than atomic displacement vectors. This geometry establishes how each mode’s Raman tensor is projected onto the local field direction.

**Figure 3:**
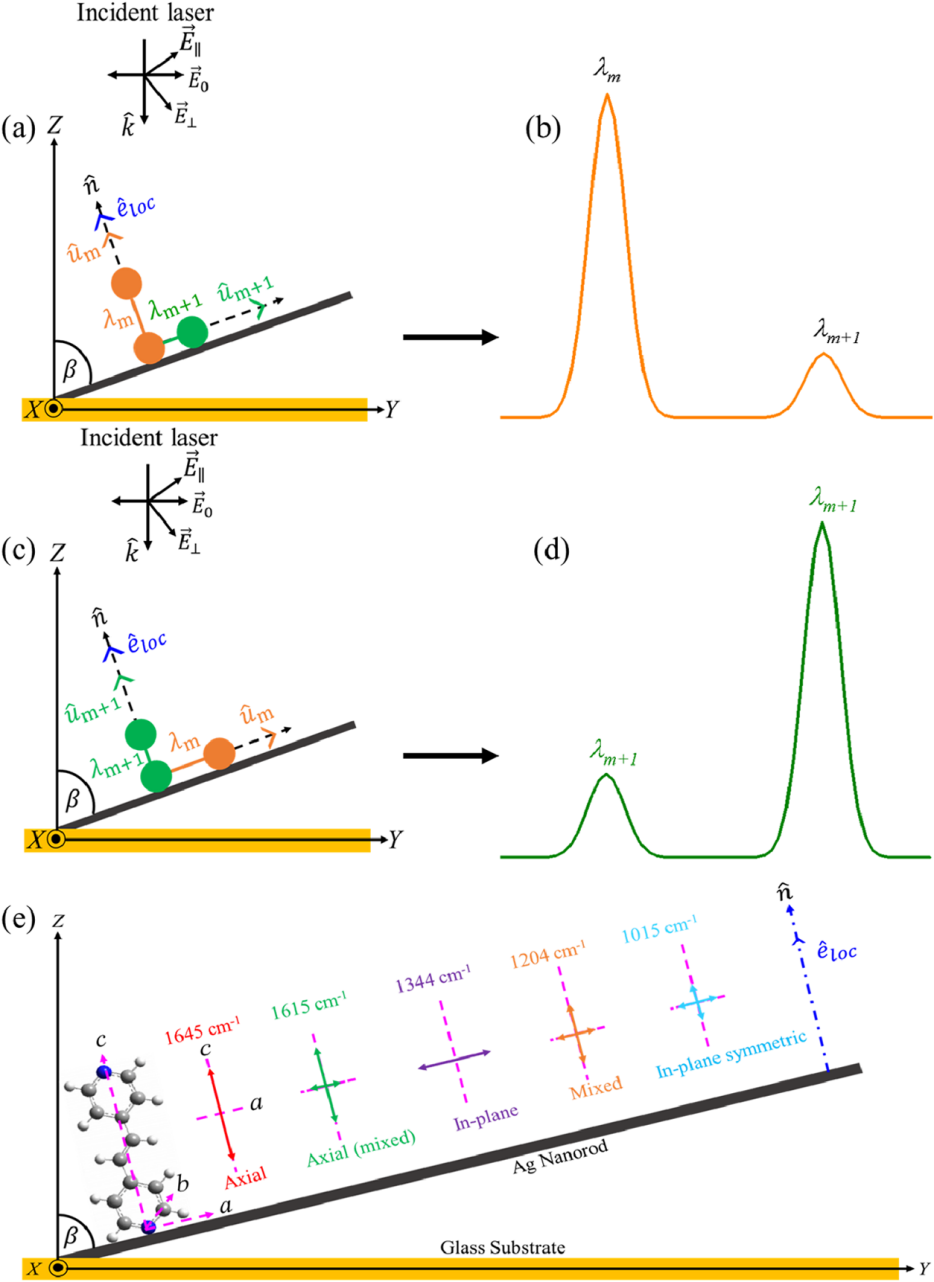
Schematic illustration of molecular orientation effect on SERS intensity for an analyte molecule adsorbed on a tilted Ag nanorod. (a) *û*_*m*_∥*n̂* and *û*_*m*+1_⊥*n̂* configuration and (b) corresponding schematic Raman spectrum dominated intensity from ν_m_. (c) *û*_*m*+1_∥*n̂* and *û*_*m*_⊥*n̂* configuration and (d) corresponding schematic Raman spectrum illustrating intensity reversal. (e) Summary of dominant polarizability-change directions for major BPE Raman modes (1645, 1615, 1344, 1204, and 1015 cm^–1^) relative to the surface-normal field on a tilted AgNR. Arrows denote the principal direction(s) of Raman polarizability change (tensor anisotropy), highlighting axial, axial-mixed, in-plane dominant, mixed, and in-plane symmetric character that governs mode-specific SERS enhancement. The molecular principal axes of adsorbed BPE are defined as *c*, long molecular axis; *a*, in-plane short axis; and *b*, out-of-plane axis normal to the molecular plane.

For metallic nanostructures such as Ag nanorods, the enhanced near field is dominated by the local surface-normal component (surface selection rule).^46,47^ We therefore approximate *ê*_local_ ≈ *n̂*. Under polarization-insensitive detection, the scattered field direction *ê*_sc_ is treated as fixed in the laboratory frame. The SERS intensity of mode *m* then reduces to

8 $${I_{m}\propto \left|{ê}_{\mathrm{sc}}\cdot {\vec{\mathrm{\alpha ⃗}}}^{\left(m\right)}\cdot \mathrm{n̂}\right|}^{2}$$

To examine orientation dependence, we consider the molecular principal-axis frame rotated relative to the local surface-normal frame. If the dominant polarizability component of a given mode lies along a molecular axis forming an angle θ_*m*_ with *n̂*, the measurable tensor projection varies approximately as

9 $${I_{m}\propto \left|\alpha_{\mathrm{eff}}^{\left(m\right)}\right|}^{2}\cos^{2}\theta_{m}$$

where α_eff_^(*m*)^ represents the effective tensor component contributing to the projection.

As illustrated in Figure 3a–d, consider two vibrational modes, λ_*m*_ and λ_*m*+1_, whose dominant polarizability directions are orthogonal. When vibrational direction *û*_*m*_ of λ_*m*_ is aligned parallel to the surface normal (θ_*m*_ = 0°) while *û*_*m*+1_ of λ_*m*+1_ is tangential (θ_*m*+1_ = 90°) (Figure 3a), the projection term maximizes *I* ^(*m*)^ and suppresses *I* ^(*m*+1)^ (Figure 3b). If molecular reorientation rotates the dominant tensor axis of *û*_*m*+1_ toward *n̂* (Figure 3c), the relative intensities invert (Figure 3d). Thus, even when electromagnetic enhancement remains unchanged, variations in adsorption geometry modify the tensor projection term in eq 7 and selectively redistribute vibrational intensities. The molecular orientation effect therefore originates directly from the anisotropic structure of the Raman polarizability tensor and its projection onto the surface-normal near field.

For BPE, we assume that one pyridyl group coordinates to the AgNR surface, aligning the molecular long axis approximately with the local surface-normal direction *n̂*, as indicated by the dash–dotted blue line in Figure 3e. Based on prior vibrational analyses and DFT calculations,^28,48–53^ the dominant Raman tensor anisotropy of the major vibrational modes can be estimated relative to *n̂*. In Figure 3e, arrows represent the principal polarizability change directions (tensor anisotropy) rather than atomic displacements. For clarity, the schematic illustrates the molecule lying within the nanorod tilting plane; however, due to the cylindrical symmetry of the AgNR surface, adsorption can occur at equivalent azimuthal orientations.

The 1645 cm^–1^ C=C stretching mode has a polarizability-change direction nearly parallel to the molecular long axis (axial dominant) and therefore couples strongly to a surface-normal field. The 1615 cm^–1^ aromatic ring stretching mode is axial-dominant but mixed, retaining a substantial surface-normal component while including secondary in-plane contributions. In contrast, the 1344 cm^–1^ ring deformation mode is predominantly in-plane dominant (tangential), with its major polarizability components lying within the molecular plane, resulting in weaker projection onto the surface-normal field. The 1204 cm^–1^ mode exhibits mixed axial and tangential character, resulting in an intermediate effective projected response. The 1015 cm^–1^ band is assigned to a totally symmetric ring-breathing vibration of BPE. Previous vibrational and DFT analyses of BPE on silver surfaces indicate that this mode exhibits a Raman tensor dominated by diagonal components with relatively similar in-plane contributions. As detailed in Section S6 of SI, under a surface-normal–dominated near field the SERS intensity is governed by the projected tensor component along the local normal direction. In contrast to the highly directional 1645 cm^–1^ C=C stretching mode, whose polarizability change is concentrated along the molecular long axis, the 1015 cm^–1^ ring-breathing mode exhibits more distributed tensor components. Consequently, its projected surface-normal response varies more gradually with molecular tilt. We emphasize that the 1015 cm^–1^ band is not orientation-invariant; rather, it is comparatively less sensitive to tilt under ensemble-averaged adsorption conditions on the AgNR substrate (see Section S6 for tensor analysis).

### BPE SERS Spectra from Six Experiments

Figure 4 shows representative BPE SERS spectra collected under six experimental conditions: defect mapping, batch-to-batch variation, nanorod length tuning, drop-cast deposition, static immersion, and real-time immersion. Across all data sets, the characteristic BPE Raman bands at 1015, 1204, 1344, 1615, and 1645 cm^–1^ are consistently observed, confirming the chemical identity and successful adsorption of BPE on AgNR substrates.^53^ However, the relative intensities of the two high-frequency bands at 1615 and 1645 cm^–1^ (denoted as *I* ^(1615)^ and *I* ^(1645)^) show clear and systematic variations across experimental conditions.

**Figure 4:**
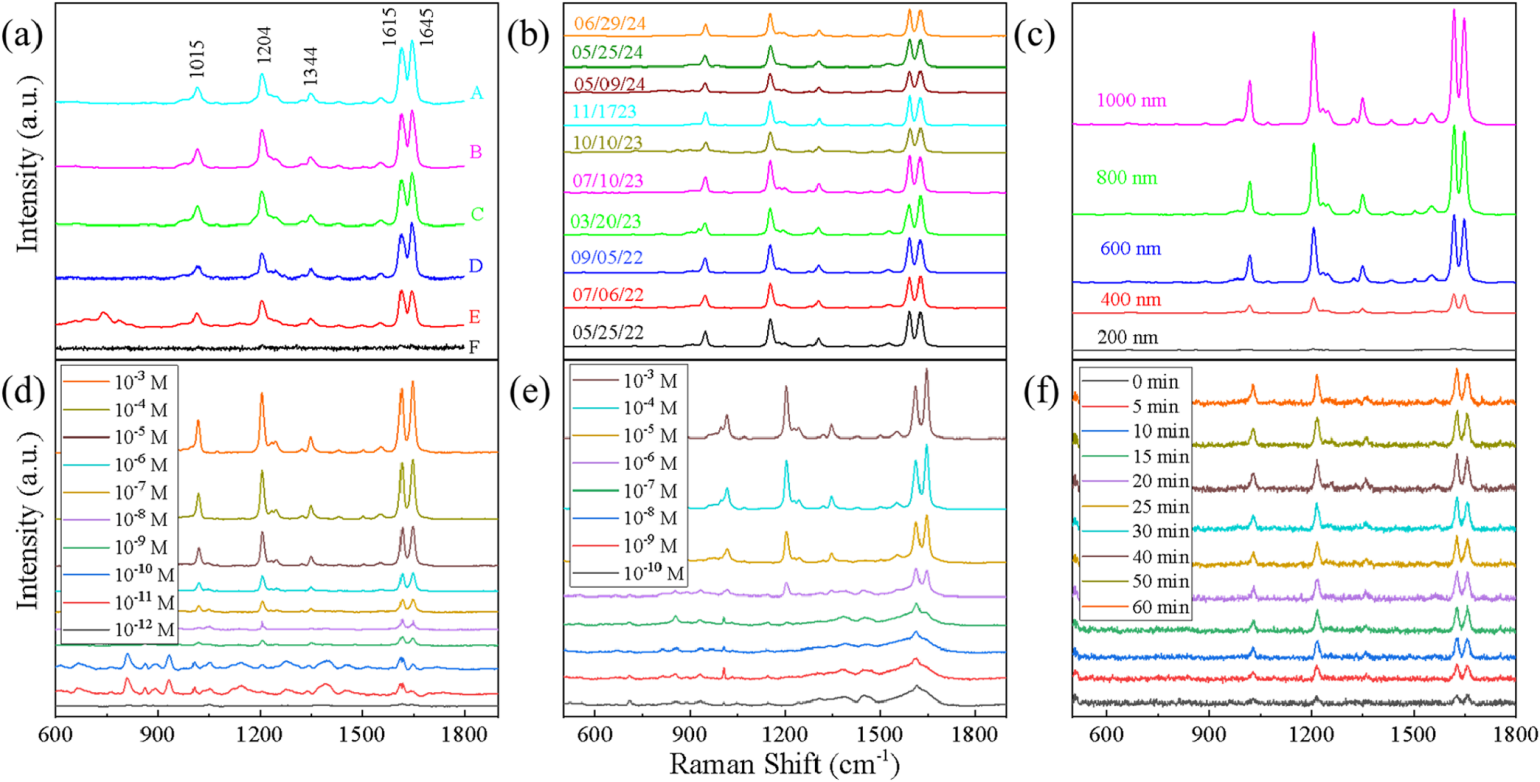
Representative SERS spectra of BPE obtained under six experimental conditions: (a) defect mapping (spectra from six different locations), (b) different AgNR batches, (c) varied nanorod lengths, (d) drop-cast adsorption with varying BPE concentrations, (e) static immersion adsorption at different BPE concentrations, and (f) time-dependent spectra for real-time immersion.

Spectra collected across a surface defect reveal clear spatial dependence (Figure 4a). The optical image of the AgNR defect and the corresponding SERS intensity heat map based on the 1204 cm^–1^ BPE band are shown in Figure S4 of SI. The defect center (location F, see Figure S4 in SI) shows no BPE features, indicating negligible SERS enhancement. Locations D and E, at the defect edge, produce measurable signals: at location E, *I* ^(1615)^ ≈ *I* ^(1645)^, while at location D, *I* ^(1615)^ < *I* ^(1645)^, suggesting field distortion and altered molecular alignment near the irregular surface. At locations A–C, farther from the defect, spectra become stronger and sharper, and the *I* ^(1615)^/*I* ^(1645)^ < 1 trend stabilizes, indicating recovery of normal enhancement. These spatial trends indicate that local morphology could strongly govern the near-field direction and magnitude.

For batch-to-batch variation (Figure 4b), all ten AgNR batches exhibit reproducible BPE signatures, but the *I* ^(1615)^/*I* ^(1645)^ ratio varies systematically among batches. Some early batches (05/25/22) show *I* ^(1615)^ > *I* ^(1645)^, others (07/06/22) yield *I* ^(1615)^ ≈ *I* ^(1645)^, and several later batches (03/20/23 and 07/10/23) display*I* ^(1615)^ < *I* ^(1645)^. These alternating trends likely arise from small fluctuations in deposition rate, film density, or nanorod morphology, which could subtly modify the substrate’s optical response and alter the relative enhancement of the two modes.

Spectra collected from nanorod arrays of different lengths (200–1000 nm) (Figure 4c) show strong geometry dependence. At length of 200 nm, overall SERS intensity is very weak. At 400 nm, signal strength increases markedly and *I* ^(1615)^ ≈ *I* ^(1645)^. For longer rods (660–1000 nm), *I* ^(1615)^ becomes progressively stronger relative to *I* ^(1645)^, yielding an increasing *I* ^(1615)^/*I* ^(1645)^ ratio. This continuous shift is consistent with the red-shift of LSPR with increasing rod length,^54^ which could modulate the field distribution and resonance overlap with the excitation and scattered wavelengths.

For drop-cast BPE (10^–3^–10^–12^ M) (Figure 4d), the *I* ^(1615)^/*I* ^(1645)^ ratio evolves with surface coverage. At higher concentrations (10^–3^–10^–5^ M), *I* ^(1645)^ > *I* ^(1615)^. At 10^–6^ M, *I* ^(1615)^ ≈ *I* ^(1645)^, and with further dilution, *I*_1615_ increasingly dominates. At the lowest concentrations (10^–11^–10^–12^ M), only the 1615 cm^–1^ band is clearly detectable. This monotonic and systematic increase in *I* ^(1615)^/*I* ^(1645)^ with BPE concentration implies a potential adsorption orientation change of BPE molecule on AgNR from low coverage to high coverage.

Static immersion produces smoother, more reproducible concentration-dependent trends (Figure 4e). At high concentrations (10^–3^–10^–5^ M), *I* ^(1645)^ > *I* ^(1615)^. As concentration decreases, *I* ^(1645)^ weakens until *I* ^(1615)^ ≈ *I* ^(1645)^ (10^–7^–10^–8^ M), and at lower concentrations, *I* ^(1615)^ > *I* ^(1645)^, eventually leaving only the 1615 cm^–1^ band visible. This trend mirrors that of the drop-cast samples and again suggests a coverage-dependent molecular orientation effect.

Time-resolved SERS spectra (0–60 min, 10^–5^ M BPE, Figure 4f) illustrate dynamic adsorption behavior. Initially, the signals are weak and *I* ^(1615)^ ≈ *I* ^(1645)^. As adsorption progresses, all bands intensify and *I* ^(1615)^ > *I* ^(1645)^ becomes more prominent, possibly reflecting molecular reorientation on the surface.

Overall, three characteristic behaviors emerge across all measurements in Figure 4: (1) noise-dominated spectra at defect regions (Figure 4a), (2) 1615 cm^–1^ band dominated spectra at low molecular coverage (Figure 4d,e), and (3) spectra that display clear BPE vibrational features but exhibit systematic variations in the *I* ^(1615)^/*I* ^(1645)^ ratio. These behaviors could originate from two intertwined physical contributions as discussed in Figures 2 and 3: Molecular orientation effects may dominate in defect mapping, drop-cast, and immersion experiments, where changes in local surface environment or surface coverage alter the alignment of BPE molecules and thus reshape the balance between *I* ^(1615)^and *I* ^(1645)^. In contrast, substrate optical effects, including variations in batch-to-batch morphology and nanorod length, could govern changes in near-field enhancement patterns that modify the relative intensities of the two high-frequency modes. More explanation will be given in the following section.

### HCA-Based Clustering

To objectively organize the spectral data set in Figure 4, we applied HCA for two purposes: (1) to separate high-signal-to-noise ratio (SNR) spectra from noisy or low-intensity spectra, and (2) to identify recurrent spectral subtypes within each quality class, enabling comparison of SERS behavior across the diverse experimental conditions. As shown in the dendrogram of Figure 5a, HCA produced a robust top-level split dividing the data set into two major branches. The upper branch contains spectra with weak intensities, poorly resolved peaks, or elevated noise levels collectively labeled as “Low-SNR” (Clusters I–III). The lower branch contains spectra with well-defined Raman features and high peak-to-noise ratio, forming the “High-SNR” group (Clusters IV–VII). To select the optimal number of clusters, both variance-based (elbow method) and separation-based (silhouette analysis) criteria were evaluated (Figure S5, Supporting Information). For the High-SNR branch, the elbow curve shows a gradual flattening of within-cluster variance reduction across *K* = 2–4, indicating that several cluster numbers in this range yield comparably reasonable partitions rather than a single well-defined optimum. The silhouette score is highest at *K* = 2, reflecting a coarse separation dominated by overall intensity differences, then decreases at *K* = 3 and exhibits a weaker local maximum at *K* = 4–5. Because solutions with *K* ≥ 5 are inconsistent with external experimental knowledge and introduce oversegmentation, *K* = 4 is selected as a balanced and interpretable compromise that increases structural resolution relative to *K* = 2–3 while avoiding excessive fragmentation. For the Low-SNR branch, the elbow analysis shows a pronounced turning point at *K* = 3, indicating a meaningful reduction in within-cluster variance at this scale. The silhouette score is highest at *K* = 2, while values for *K* = 3–5 are similarly lower (approximately 0.18), reflecting limited separability under noise-dominated conditions. This combination of diagnostics supports *K* = 3 as a reasonable and interpretable clustering choice. Overall, these results illustrate the trade-off between maximizing numerical separation and preserving physically meaningful structure, motivating the selection of 4 subclusters for the High-SNR branch and 3 subclusters for the Low-SNR branch. The selection reflects the natural structure of the data set rather than predetermined experimental labels. The heat map in Figure 5a (right panel) shows consistent BPE vibrational modes (1015, 1204, 1344, 1615, 1645 cm^–1^) as vertical streaks, while differences in their relative intensities and noise levels distinguish the subclusters. The metadata bar (between the dendrogram and the heat map) maps each spectrum to its experimental origin. Although clustering was performed entirely unsupervised, certain experimental conditions tend to populate particular clusters, reflecting how physical factors substrate structure, adsorption environment, and local hotspot quality manifest in spectral patterns.

**Figure 5:**
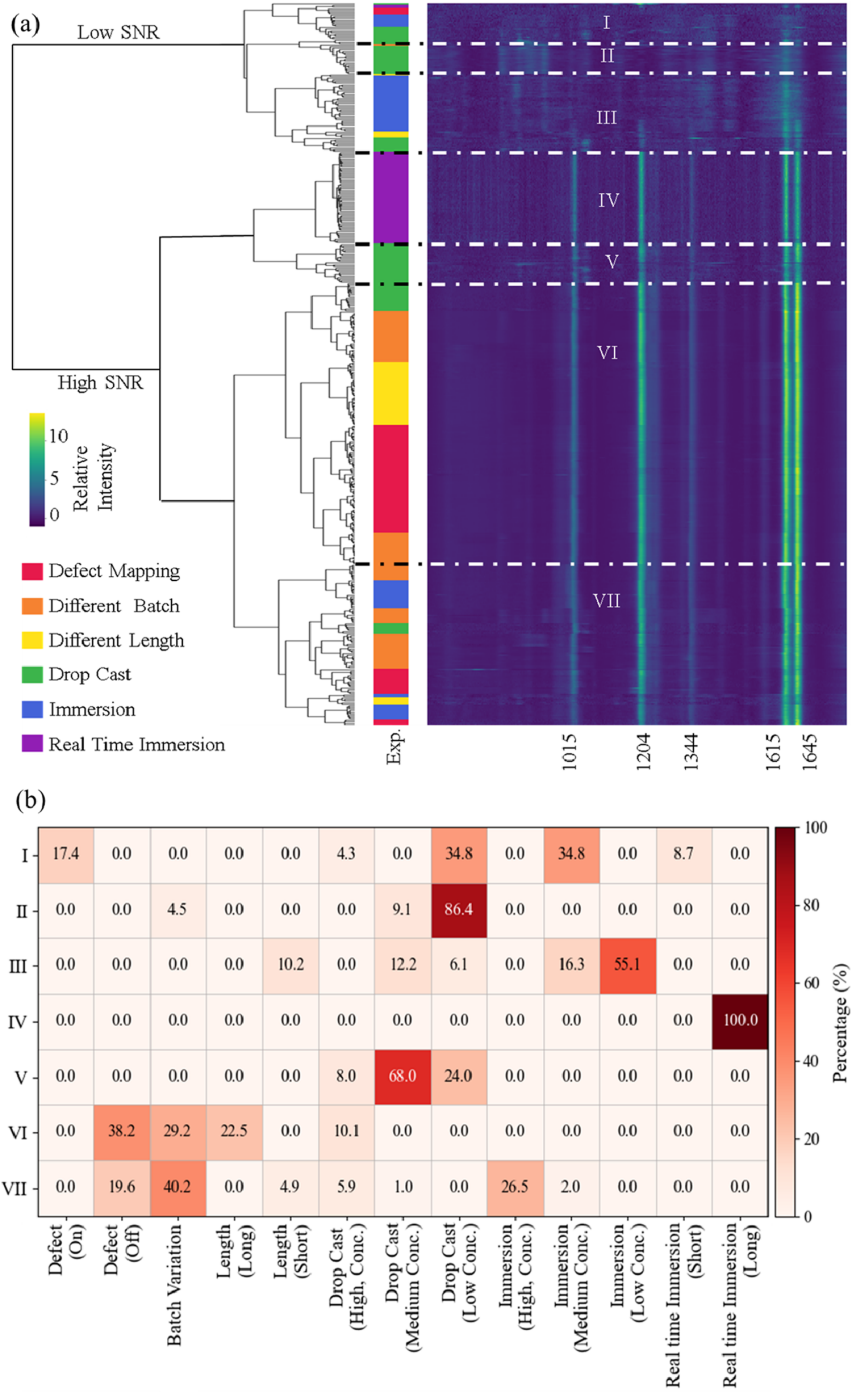
(a) HCA of all BPE SERS spectra collected across six different experimental conditions. The dendrogram (left) revealing a clear separation between Low-SNR and High-SNR populations. The heat map (right) displays normalized relative intensity as a function of Raman wavenumber, with warmer colors indicating higher intensity. (b) Percentage of spectra from different experimental conditions contributes to different clusters.

To further quantify these relationships, we analyzed the fractional contributions of different experimental conditions to each cluster, based on the metadata defined in the Methods section and the total number of spectra assigned to each cluster. Figure 5b shows the cluster-normalized (row-wise) distribution, illustrating how spectra from different experimental conditions contribute to each HCA-defined cluster. This statistical breakdown establishes a direct link between the unsupervised spectral clusters and the underlying experimental variables and is discussed together with the corresponding cluster-averaged spectral shapes in the following sections.

Figure 6 presents the average spectrum and corresponding ±1σ variation for each of the 7 clusters identified by HCA. Cluster I (Figure 6a) exhibits a very low SNR ratio. Although the five characteristic BPE bands at 1015, 1204, 1344, 1615, and 1645 cm^–1^ are still discernible, they are superimposed on a strong background with large spectral fluctuations. The intensity variations are particularly pronounced at the BPE peak positions, indicating unstable or weak enhancement. As indicated in Figure 5b, Cluster I is dominated by spectra from low-concentration drop-cast experiments (10^–9^–10^–12^ M, 34.8%), medium-concentration immersion experiments (10^–6^–10^–8^ M, 34.8%), and defect mapping measurements taken on or near surface defects (17.4%). Early time real-time immersion spectra (<5 min, 8.7%) contribute only a small fraction. These results indicate that Cluster I primarily reflects conditions with insufficient molecular coverage or poor local electromagnetic enhancement.

**Figure 6:**
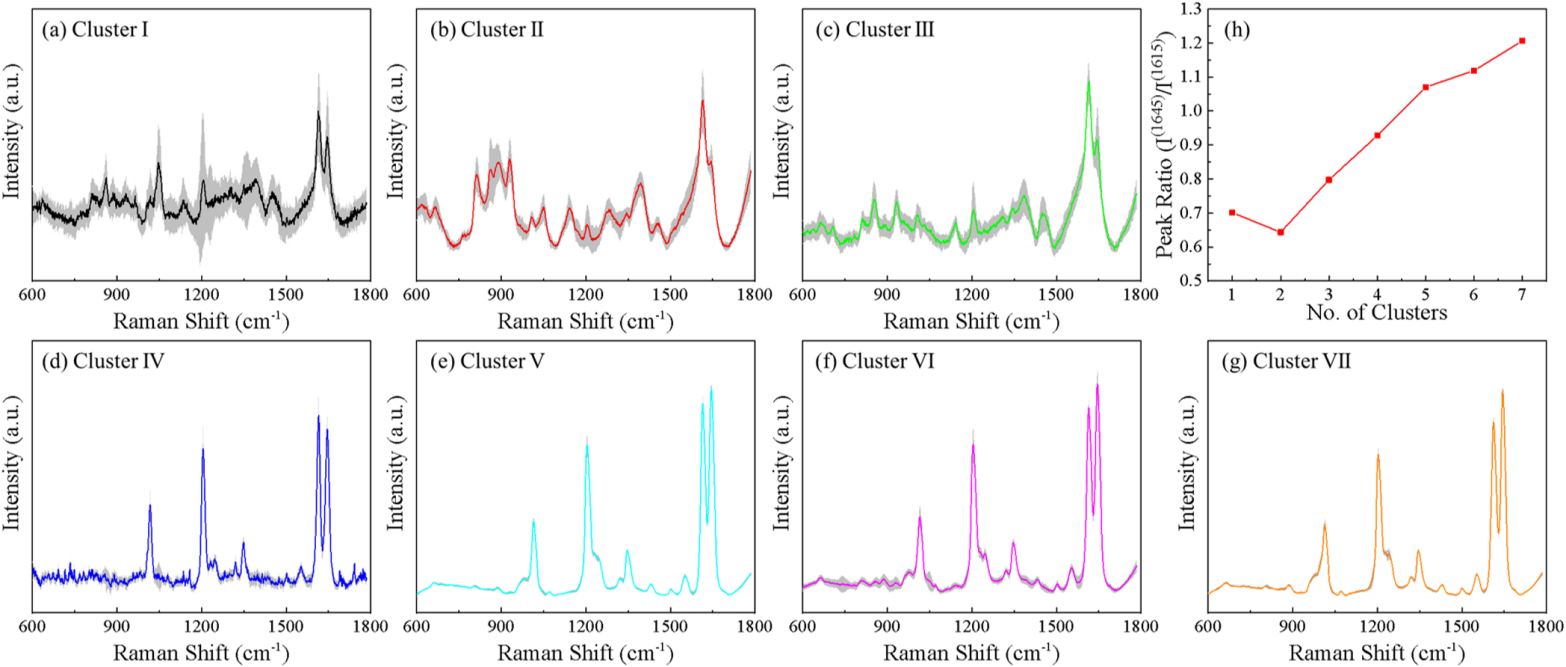
Average and variations of BPE spectra for 7 clusters: (a–c) the Low-SNR Clusters I–III; (d–g) the High-SNR Clusters IV–VII. (h) The progressive change of *I* ^(1645)^/*I* ^(1615)^ ratio for the 7 clusters.

The average spectra of Cluster II (Figure 6b) and Cluster III (Figure 6c) show similar Low-SNR characteristics but with distinct background features. In both clusters, the 1615 and 1645 cm^–1^ bands are broadened and partially merged, while the lower-frequency BPE modes at 1015, 1204, and 1344 cm^–1^ are weak or barely resolved. In addition, prominent and cluster-specific background features appear in the 700–1500 cm^–1^ region, indicating different sources of nonanalyte contributions. According to Figure 5, Cluster II is primarily populated by low-concentration drop-cast spectra (10^–9^–10^–12^ M, 86.4%). Minor contributions arise from medium-concentration drop-cast measurements (10^–6^–10^–8^ M, 9.1%) and batch-to-batch variation effects (4.5%). Cluster III shows a similar spectral shape to Cluster II in wavenumber range above 1500 cm^–1^, but resembles Cluster I in the 600–1500 cm^–1^ range. It is dominated by low-concentration immersion spectra (10^–9^–10^–10^ M, 55.1%). Cluster III also receives significant contributions from medium-concentration immersion experiments (10^–6^–10^–8^ M, 16.3%) and low and medium-concentration drop-cast measurements (10^–6^–10^–12^ M, 18.3%), as well as substrates with short nanorod lengths (200 nm, 10.2%). Notably, for all Low-SNR clusters (I–III), the intensity ratio *I* ^(1645)^/*I* ^(1615)^ is always less than 1.

In contrast, the High-SNR clusters (Clusters IV–VII, Figure 6d–g) exhibit highly consistent and well-defined spectral signatures. In all cases, the five characteristic BPE bands at 1015, 1204, 1344, 1615, and 1645 cm^–1^ are clearly resolved, while background contributions and noise are strongly suppressed. Only Cluster IV (Figure 6d) shows a modest residual noise level, whereas Clusters V–VII display nearly noise-free average spectra. Correspondingly, the spectral variations across these clusters are minimal, indicating highly reproducible and intrinsically high-quality SERS measurements. A clear distinction among the High-SNR clusters is observed in the relative intensities of the two high-frequency modes. Specifically, $\frac{I^{\left(1645\right)}}{I^{\left(1615\right)}}<1$ for Cluster IV, whereas $\frac{I^{\left(1645\right)}}{I^{\left(1615\right)}}>1$ for Clusters V–VII, reflecting systematic differences in molecular orientation and/or local field distribution. Figure 6h plots *I* ^(1645)^/*I* ^(1615)^ as a function of cluster index and shows a clear monotonic increase, demonstrating both the robustness of the HCA classification and a systematic evolution of BPE spectral characteristics driven by relative peak intensity changes. Based on the experimental metadata (Figure 5b), Cluster IV consists almost exclusively of real-time immersion spectra (10^–5^ M, ∼100%), suggesting equilibrium adsorption conditions that favor enhanced coupling to the 1615 cm^–1^ mode. Cluster V receives major contributions from medium-concentration drop-cast experiments (10^–6^–10^–8^ M, ∼68%) and additional contributions from low-concentration drop-cast measurements (10^–9^–10^–12^ M, ∼24%) suggest partial overlap with weaker adsorption environments, while only a minor fraction originates from high-concentration drop-cast experiments (10^–3^-10^–5^ M, ∼8%). Cluster VI is primarily dominated by spectra acquired away from defect regions (defect-off, 38.2%). Substantial contributions also arise from batch-to-batch variation experiments (∼29.2%) and nanorod-length-dependent measurements using longer nanorods (400–1000 nm, ∼22.5%). A smaller but notable fraction of spectra originates from high-concentration drop-cast measurements (10^–3^-10^–5^ M, ∼10.1%). Similarly, Cluster VII is dominated by batch-to-batch variation effects (∼40.2%), indicating strong sensitivity to substrate fabrication differences. This cluster also shows a substantial contribution from high-concentration immersion measurements (10^–3^–10^–5^ M, ∼26.5%), followed by spectra collected away from defect regions (defect-off, ∼19.6%). Smaller contributions arise from high-concentration drop-cast experiments (∼5.9%) and short nanorod length substrates (200 nm, ∼4.9%). These diverse contributions indicate that Clusters VI and VII represent spectra acquired under optimal enhancement conditions across multiple experimental configurations.

Figure 7 presents the complementary condition-normalized (column-wise) distribution, showing how spectra from a given experimental condition redistribute across the seven HCA-defined clusters, providing a complementary view from the experimental-condition perspective. From this condition-normalized perspective, several systematic trends emerge. First, some conditions map uniquely to a single cluster: spectra acquired on defects and early time real-time immersion (<5 min) contribute exclusively to Cluster I; low-concentration immersion (10^–9^–10^–10^ M) spectra map entirely to Cluster III; long-time real-time immersion spectra populate Cluster IV; spectra from long nanorods (400–1000 nm) map exclusively to Cluster VI; and high-concentration immersion (10^–3^–10^–5^ M) spectra contribute entirely to Cluster VII. Second, several experimental conditions distribute across two High-SNR clusters. Spectra acquired away from defect regions (defect-off), those associated with batch-to-batch variation, and high-concentration drop-cast (10^–3^–10^–5^ M) measurements contribute predominantly to Clusters VI and VII, reflecting consistently strong electromagnetic enhancement and reliable BPE distribution among hotspots under these conditions. In contrast, medium-concentration immersion (10^–6^–10^–8^ M) and low-concentration drop-cast (10^–9^–10^–12^ M) spectra populate mainly Clusters I–III, indicating a strong influence of low surface coverage and high fluctuation in enhancement. Third, some conditions split between Low- and High-SNR clusters, indicating borderline regimes. Medium-concentration drop-cast (10^–6^–10^–8^ M) spectra show a mixed distribution, with ∼65.4% assigned to Cluster V and ∼30% to Clusters II and III, highlighting the sensitivity of static drop-casting to local adsorption and hotspot variability. Similarly, spectra from long nanorods (400–1000 nm) split between Cluster III (50%) and Cluster VII (50%), showing the sensitivity of spectral shape to the optical response of the substrates.

**Figure 7:**
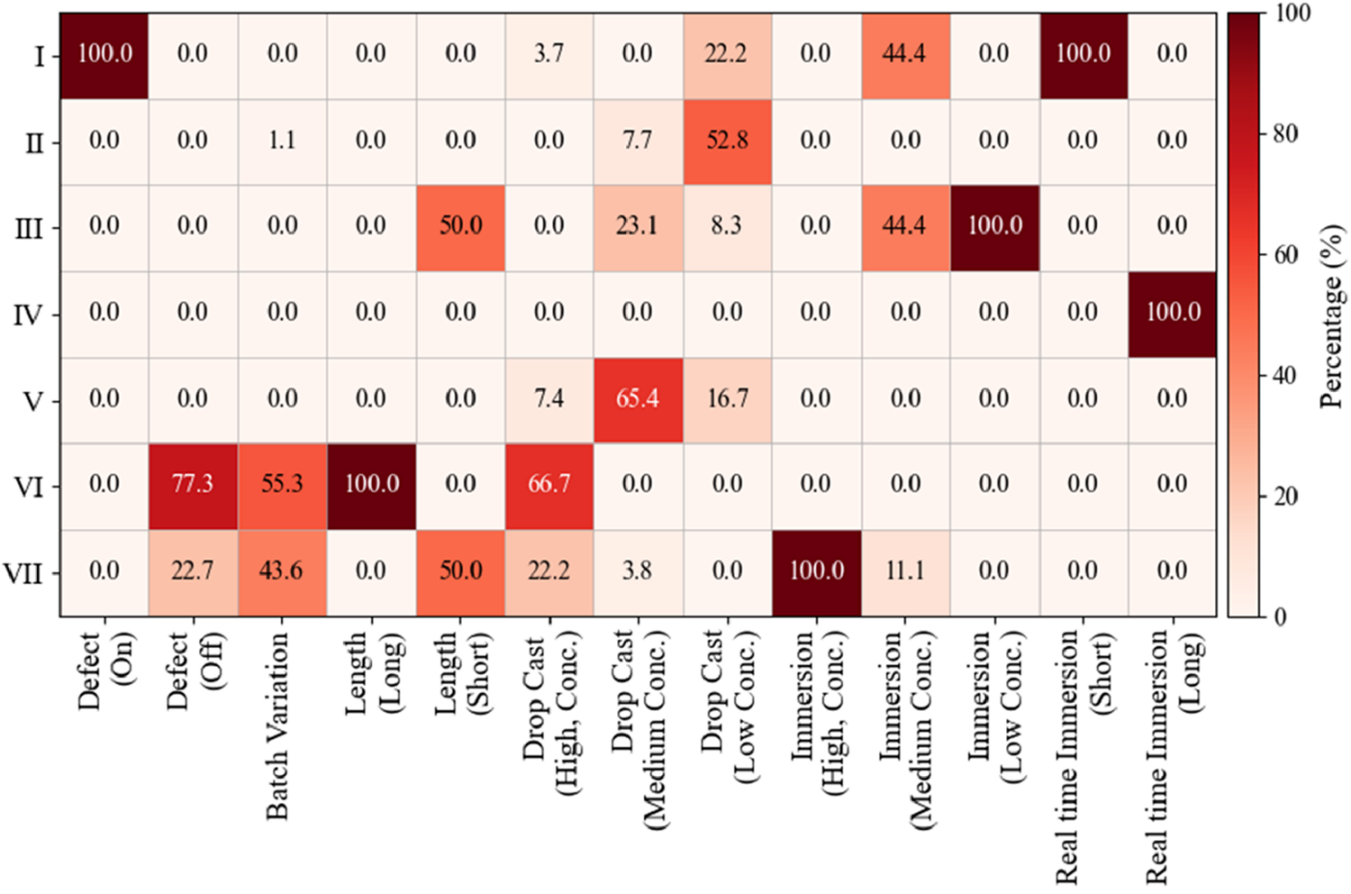
Redistribution of SERS spectra from different experimental conditions across the seven HCA-defined clusters.

Taken together, these distributions clarify the physical origins of the clusters. The Low-SNR clusters (I–III) arise from two dominant factors: (i) insufficient analyte signal, due to low molecular coverage or early adsorption stages, which allows noise and background contributions to dominate; and (ii) weak electromagnetic enhancement, associated with surface defects or short nanorods, which limits SERS gain even when analyte molecules are present. These effects produce poorly resolved BPE features and large spectral variability, likely due to biased molecular adsorption orientations. In contrast, the defining characteristics of the High-SNR clusters (IV–VII), particularly Clusters VI and VII, are high analyte concentration and strong, spatially uniform electromagnetic enhancement, enabled by high-quality Ag nanorod substrates and well-developed SERS hot spots. Under these conditions, intrinsic BPE vibrational features dominate the spectra, noise and background are minimized, and systematic variations in *I* ^(1645)^/*I* ^(1615)^ primarily reflect controlled changes in molecular orientation and substrate optical response rather than measurement artifacts. Clusters IV and V, however, occupy intermediate regimes: Cluster IV is highly sensitive to adsorption dynamics under equilibrium immersion, while Cluster V reflects coverage-dependent variability under static drop-cast conditions, making both clusters particularly responsive to changes in BPE surface coverage and molecular reorientation rather than to substrate defects or noise.

### Quantitative Comparison of Vibrational-Mode Intensities

To gain further insight into the spectral organization revealed by HCA, we performed PCA on the spectra from all seven clusters (Figure 8). In the PC1–PC2 score plot (Figure 8a), the clusters form largely separated groups, with only limited overlap at their boundaries. This independent dimensionality-reduction analysis further supports the robustness and physical relevance of the HCA-based classification. The PC1 loading curve (Figure 8b) reveals the dominant spectral features responsible for cluster separation. Notably, the loading profile closely mirrors the intrinsic BPE vibrational spectrum, with the largest absolute loadings occurring at the five characteristic BPE bands at 1645 > 1204 > 1615 > 1015 > 1344 cm^–1^. This indicates that the primary variance captured by PC1 originates from systematic changes in the relative intensities of these BPE modes, rather than from random noise or background fluctuations. In particular, the strong loadings at 1615 and 1645 cm^–1^ are consistent with the cluster-dependent evolution of the *I* ^(1645)^/*I* ^(1615)^ ratio identified in the HCA results.

**Figure 8:**
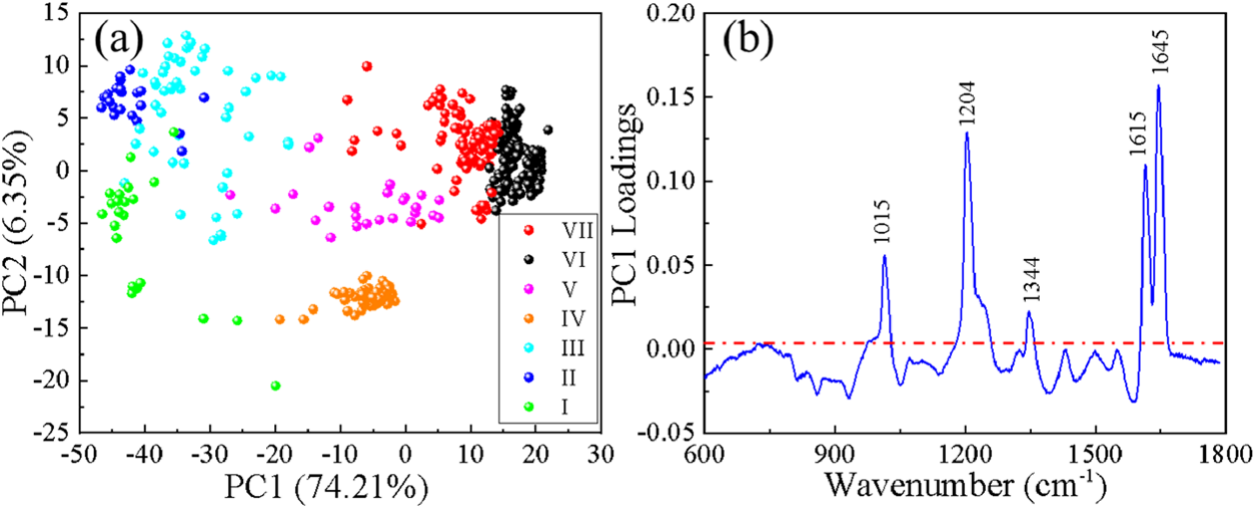
PCA of BPE SERS spectra of the seven clusters. (a) The PC1-PC2 score plot. (b) PC1 loading curve plot.

Guided by the combined HCA and PCA results, we extracted the intensities of the five dominant BPE modes (1645, 1615, 1344, 1204, and 1015 cm^–1^) for each of the seven clusters. Although Figure 6h highlights only the *I* ^(1645)^/*I* ^(1615)^ ratio as a convenient descriptor, the PCA loading analysis (Figure 8b) demonstrates that spectral classification is governed by the relative intensity ratios among all five modes. To separate physically meaningful trends from noise and global intensity scaling, especially in the Low-SNR clusters, we constructed intensity web plots using the cluster-averaged spectra from Figure 6 and applied three complementary normalization strategies, each designed to emphasize a distinct physical contribution to the observed SERS response. To clarify the physical motivation of each strategy, Table 1 summarizes the normalization reference, the physical contribution emphasized by each normalization strategy used in Figure 9.

**Figure 9:**
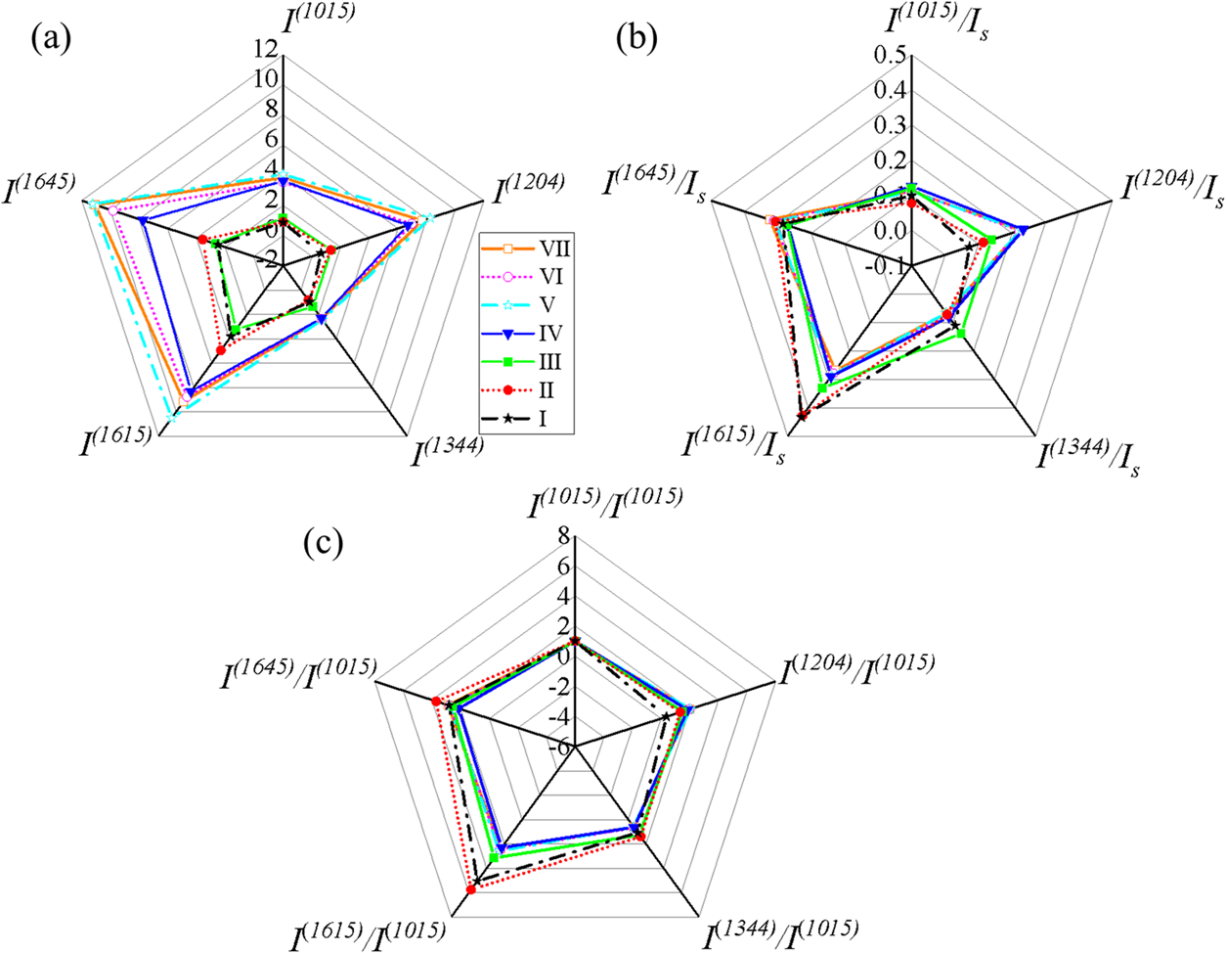
Web plot of 5 characteristic modes of BPE from 7 clusters. (a) Intensity plot extracted from Figure 6; (b) normalized by *I*_*s*_; and (c) normalized by *I* ^(1015)^.

**Table 1:** Summary of Normalization Strategies Used for Quantitative Comparison of BPE Vibrational-Mode Intensities

normalization strategy	normalization reference	physical contribution emphasized
Absolute intensities (Figure 9a)	None (raw peak intensities)	Global electromagnetic enhancement amplitude and effective analyte coverage
Sum-normalized intensities (Figure 9b)	Total intensity of five BPE modes	Wavelength-dependent electromagnetic reweighting of Raman modes (suppresses global scaling; highlights spectral-shape redistribution)
Mode-referenced normalization (Figure 9c)	1015 cm^–1^ BPE ring-breathing mode	Relative Raman tensor projection effects (orientation-dependent mode-to-mode comparison; global EM scaling reduced but not eliminated)

Figure 9a compares the absolute intensities of the five BPE modes across clusters. For Low-SNR clusters (I–III), direct peak extraction is unreliable due to peak broadening and noise; Gaussian fitting was therefore used to recover peak amplitudes. The resulting web plot primarily reflects the prominence of the five characteristic peaks. Clusters I–III exhibit similarly small enclosed areas, markedly smaller than those of Clusters IV–VII. In contrast, the High-SNR clusters show much larger enclosed areas with a clear progression in overall intensity (Cluster IV < V < VI < VII), indicating increasing electromagnetic enhancement and/or analyte coverage.

To suppress global intensity differences, each mode intensity *I* ^(*m*)^ was normalized by the sum of the five peak intensities, *I*_*s*_ = ∑*_m_I* ^(*m*)^, with *m* = 1645, 1615, 1344, 1204, and 1015 cm^–1^. This normalization highlights how intensity is redistributed among vibrational modes, emphasizing spectral shape rather than amplitude. As shown in Figure 9b, all seven clusters exhibit distinct shapes, with the largest differences observed among Clusters I–IV. In contrast, Clusters V–VII display very similar shapes, indicating that once strong SERS enhancement is achieved, substrate optical effects play a limited role in modifying the relative spectral profile. For Low-SNR spectra, however, both background contributions and molecular orientation strongly influence the apparent spectral shape.

All peak intensities were additionally normalized to the 1015 cm^–1^ band (*I* ^(1015)^) to provide an internal reference for mode-to-mode comparison. We emphasize that this normalization does not eliminate wavelength-dependent electromagnetic weighting, since the enhancement factor remains dependent on both excitation and scattered wavelengths. However, because the Ag nanorod substrates exhibit a broadband and smoothly varying plasmonic response across the 1000–1700 cm^–1^ Raman window under 785 nm excitation (Section S2 of SI), the electromagnetic weighting term varies gradually across these modes rather than exhibiting sharp resonance-driven selectivity. Under the surface-normal–dominated near field condition of AgNR substrates (Figure 3e; Section S6), pronounced deviations from smooth spectral trends are therefore more sensitively governed by differences in Raman tensor projection (i.e., molecular orientation effects) than by abrupt optical resonance shifts. For the High-SNR clusters (V–VII), the resulting web plots show highly consistent shapes, consistent with the behavior observed in Figure 9c. In contrast, the Low-SNR clusters (I–III) exhibit large and systematic variations. Specifically, the ratios *I* ^(1615)^/*I* ^(1015)^ and *I* ^(1645)^/*I* ^(1015)^ are larger for Clusters I–III than for Clusters IV–VII, whereas *I* ^(1204)^/*I* ^(1015)^ is smaller. The ratio *I* ^(1615)^/*I* ^(1015)^ exhibits pronounced fluctuations, with Cluster II showing the largest value and Cluster III the smallest. Within the Low-SNR regime, substantial cluster-to-cluster variation is also observed. For *I* ^(1615)^/*I* ^(1015)^ and *I* ^(1645)^/*I* ^(1015)^, the ordering follows Cluster II > Cluster I > Cluster III > Cluster IV–VII. For *I* ^(1204)^/*I* ^(1015)^, the trend reverses to Cluster IV–VII > Cluster III > Cluster II > Cluster I, while for *I* ^(1344)^/*I* ^(1015)^, the ordering is Cluster II > Cluster I–Cluster III > Cluster IV–VII. Taken together with the orientation dependence summarized in Figure 3e, these results indicate that Low-SNR spectra more susceptible to apparent orientation-dependent variations due to reduced electromagnetic enhancement and increased adsorption heterogeneity, whereas High-SNR spectra are dominated by robust electromagnetic enhancement with relatively stable molecular configurations.

## Conclusion

This work provides a comprehensive and systematic investigation of SERS spectral shape variability using BPE adsorbed on geometrically random AgNR substrates as a model system. By combining carefully designed experiments with unsupervised chemometric analysis, we demonstrate that SERS spectral shape variations are not random artifacts but arise from well-defined and physically interpretable mechanisms involving substrate optical response, electromagnetic enhancement strength, molecular adsorption orientation, and experimental conditions. The resulting data set, spanning six complementary experimental conditions, including defect mapping, batch-to-batch variation, nanorod length tuning, concentration-controlled drop-casting, static immersion, and real-time immersion, captures a wide range of realistic SERS measurement environments. A key outcome of this study is the demonstration that HCA, when applied carefully and interpreted in conjunction with physical metadata, provides a powerful and objective means to organize complex SERS data sets. Rather than presupposing experimental labels or relying on signal intensity thresholds alone, HCA reveals the natural structure of the data, separating spectra into Low- and High-SNR regimes and further resolving reproducible spectral subtypes within each regime. Importantly, this clustering reflects genuine physical differences, such as surface defects, enhancement quality, analyte coverage, and adsorption dynamics, rather than arbitrary statistical partitioning. This highlights the importance of cautious cluster-number selection, validation using complementary metrics, and physical interpretation of cluster membership, rather than treating clustering as a purely algorithmic step.

Beyond clustering, this work underscores the limitations of relying on single peak ratios, such as the commonly used *I* ^(1645)^/*I* ^(1615)^ ratio, for interpreting SERS spectral variability. While such ratios provide convenient descriptors, our PCA results and cluster-averaged spectral analyses clearly show that spectral classification and separation are governed by the collective redistribution of intensities across the full set of characteristic vibrational modes. The PCA loading analysis demonstrates that the dominant variance in the data set arises from correlated changes among all five intrinsic BPE modes, emphasizing that spectral shape must be understood as a multivariate feature rather than a two-peak comparison.

The combined use of HCA and PCA emerges as a particularly powerful strategy. HCA provides discrete, interpretable spectral groupings tied to experimental conditions, while PCA identifies the dominant spectral features and vibrational modes responsible for those groupings. Together, they enable a clear separation between signal-quality effects and intrinsic spectral-shape variations. This combined approach also provides internal consistency checks: the monotonic evolution of cluster-averaged peak ratios, the agreement between PCA loadings and physical vibrational assignments, and the coherence of cluster-resolved experimental metadata collectively reinforce the robustness of the interpretation.

By further integrating multiple intensity-normalization strategies through web-plot analysis, we are able to isolate different governing physical principles. Absolute intensities primarily reflect electromagnetic enhancement strength and analyte coverage, whereas normalized intensities by peak sums emphasize substrate-dependent optical reweighting, and normalization to the 1015 cm^–1^ mode highlights orientation-selective enhancement. This layered analysis leads to a clear physical picture: electromagnetic enhancement governs whether spectra enter a high-quality regime, while molecular orientation relative to the surface-normal near field determines how vibrational intensities are redistributed within that regime. Low-SNR spectra are therefore dominated by noise, background, and unstable adsorption configurations, whereas High-SNR spectra reflect robust enhancement with systematic, physically meaningful shape variations.

More broadly, the experience gained from this study suggests several practical guidelines for future SERS investigations. First, spectral variability should be treated as an informative signal rather than discarded as experimental noise. Second, whole-spectrum analysis and multivariate methods should be prioritized over isolated peak metrics. Third, unsupervised clustering should be combined with physical reasoning and experimental metadata to avoid overinterpretation. Finally, normalization strategies must be chosen deliberately, with explicit awareness of the physical contributions they emphasize or suppress.

It is important to emphasize that while real-world sensing environments may introduce additional chemical variables such as pH variation, ionic strength, competitive adsorption, and biomolecular interference, the ability to interpret such complexities depends critically on first resolving the intrinsic physical origins of spectral redistribution. Without separating electromagnetic spectral reweighting from orientation-dependent Raman tensor projection or other potential related effects, spectral differences may be misattributed to chemical effects, leading to erroneous conclusions or misleading sensing claims. The controlled framework developed here therefore serves as a mechanistic baseline rather than a fully comprehensive model of all SERS systems. By explicitly integrating multiple experimental variables within a physics-grounded interpretive structure, this study outlines a pathway toward more rigorous spectral interpretation in increasingly complex SERS environments.

In summary, this work establishes a physics-informed, data-driven framework for understanding and interpreting SERS spectral shape variations under complex experimental conditions. By integrating systematic experiments, careful clustering, multivariate analysis, and physical modeling, we provide not only mechanistic insight into SERS variability but also a transferable analytical strategy applicable to heterogeneous substrates, low-concentration sensing, and dynamic adsorption processes. These insights lay a solid foundation for more robust, interpretable, and reproducible SERS measurements across chemical, biological, and environmental sensing applications.

## Supplementary Material



## Data Availability

The data and codes supporting the main findings of this study are available from the corresponding authors upon reasonable request.

## Notes

SEM characterization and structural schematics of AgNR substrates; optical characterization (reflectance and effective extinction spectra) before and after BPE adsorption; detailed defect-mapping experiments correlating local morphology with SERS enhancement; additional hierarchical clustering analysis supporting subcluster selection using elbow and silhouette criteria for High- and Low-SNR spectral groups; metadata definitions for defect-mapping classification; and a tensor-based framework describing electromagnetic weighting and orientation-dependent Raman tensor projections for BPE on AgNR substrates (PDF)
