# Supplementary material for: Understanding SERS Spectral Shape Variability through Substrate Optics, Molecular Orientation, and Unsupervised Clustering
Source: J Phys Chem C Nanomater Interfaces. 2026 Mar 24;130(13):4852–67. doi: 10.1021/acs.jpcc.5c08676 (PMC13051446; doi:10.1021/acs.jpcc.5c08676)
Supplement: Supplementary file 1 [file jp5c08676_si_001.pdf]

Supporting Information

Understanding SERS Spectral Shape Variability through Substrate Optics, Molecular Orientation, and Unsupervised Clustering

Amit Kumar[1], Fengbo Ma[2], Xianyan Chen[3], Yiping Zhao[1*]

[1] Department of Physics and Astronomy, The University of Georgia, Athens, Georgia 30602, United States

[2] School of Electrical and Computer Engineering, The University of Georgia, Athens, Georgia 30602, United States

[3] Department of Epidemiology & Biostatistics, College of Public Health, The University of Georgia, Athens, Georgia 30602, United States

*E-mail: zhaoy@uga.edu

Table of Contents

S1. Characterization of the AgNR substrate 3

S2. Optical Characterization of Ag Nanorod Substrates 3

S3. Additional Experimental Details: Defect mapping 5

S4. Additional Hierarchical Clustering Analysis: Selecting Subclusters within High-SNR and Low-SNR Groups 6

S5. Additional information about Defect-mapping metadata 6

S6. Electromagnetic weighting and orientation-dependent Raman tensor projections for BPE on AgNR 7

S1. Characterization of the AgNR Substrate.

AgNR substrates were fabricated using oblique angle deposition (OAD) on glass substrates. The deposition was carried out at a tilt angle of 86°, producing vertically inclined nanorods with high aspect ratios and controlled inter-rod spacing[1].

Figure S1a schematically illustrates the cross-sectional structure of the AgNR substrate and defines the geometric parameters. The nanorods have length L, measured along the rod axis, and are inclined at an angle β ($\approx 72°$) relative to the substrate normal. The effective AgNR layer thickness d corresponds to the vertical projection of the nanorod array. The underlying structure consists of a 100 nm Ag film and a 10 nm Ti adhesion layer on glass. This morphology supports strong localized surface plasmon resonances and reproducible SERS enhancement. **Figure S1b** shows a representative SEM image of the surface of the AgNR substrates.

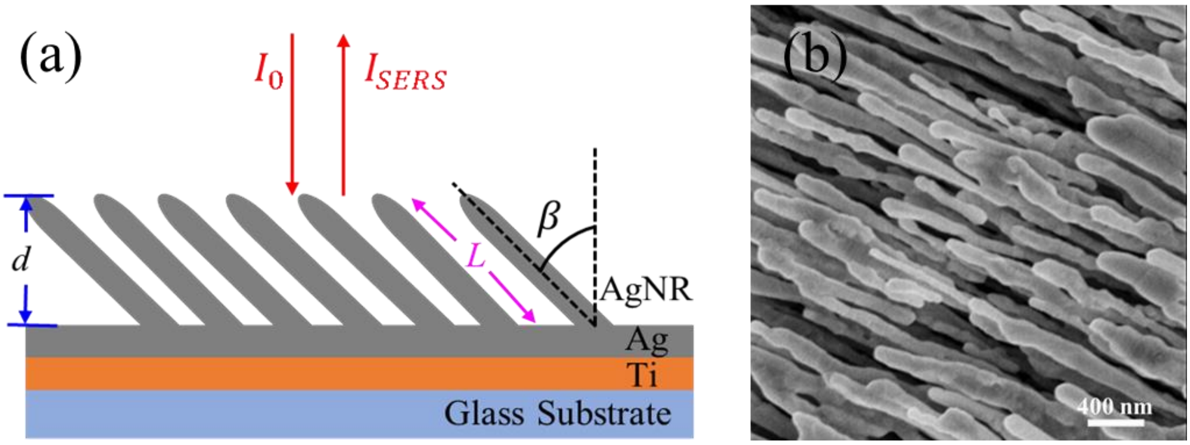

Figure S1. (a) Cross-sectional schematic of the AgNR-on-film substrate architecture, and (b) a representative top-view SEM image of AgNR arrays fabricated by OAD.

S2. Optical Characterization of Ag Nanorod Substrates

Reflectance spectra $R(\lambda)$ of AgNR substrates with lengths of 200, 400, 600, 800, and 1000 nm deposited on Ag film were measured over the visible–near-infrared range at 8° incident angle by a Shimadzu UV-2450 Spectrophotometer (**Figure S2**). Because the nanorods are grown on a thick Ag underlayer whose reflectance approaches unity in this spectral region, transmission is negligible. Therefore, reflectance provides an appropriate far-field optical characterization.

An effective extinction (i.e., effective absorbance) $A_{eff}(\lambda)$ was calculated as

$$A_{eff}(\lambda) = -\ln R(\lambda).$$

Under a double-pass approximation for AgNR-on-Ag-film architectures, this quantity can be expressed as $A_{eff}(\lambda) \approx 2\alpha(\lambda)d$, where α is an effective absorption coefficient of the AgNR layer and d is the nanorod thickness.

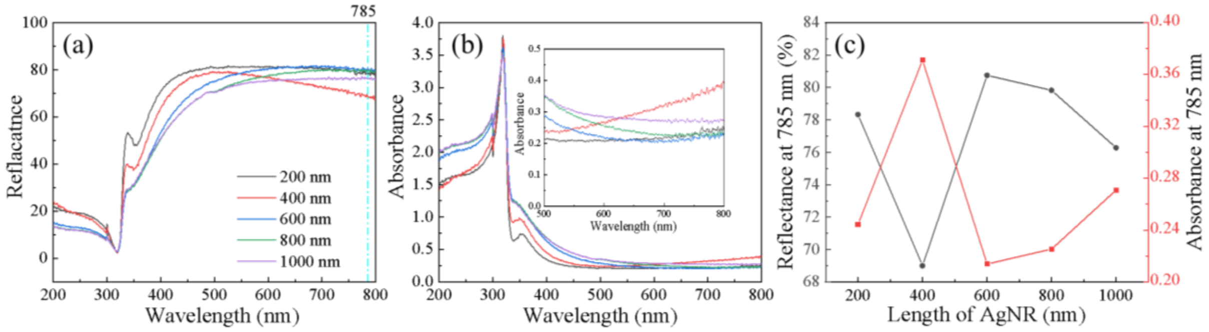

Figure S2. (a) Measured reflectance spectra of AgNR substrates with lengths of 200, 400, 600, 800, and 1000 nm deposited on an Ag film. The dashed vertical line indicates the 785 nm excitation wavelength used in SERS measurements. (b) Corresponding effective extinction spectra. (c) Extracted reflectance (left axis) and effective extinction (right axis) values at 785 nm as a function of nanorod length.

Figures S2a and **S2b** show the reflectance and corresponding effective extinction spectra for all nanorod lengths. In both cases, the spectra exhibit a broadband optical response, without the emergence of a distinct, length-dependent LSPR peak. At the 785 nm excitation wavelength used in SERS measurements, the reflectance varies between ~69–81% (**Figure S2c**), corresponding to effective extinction values of ~0.21–0.36 across the tested lengths. These values are comparable to those reported for discretized nanostructures exhibiting LSPR behavior[2, 3].

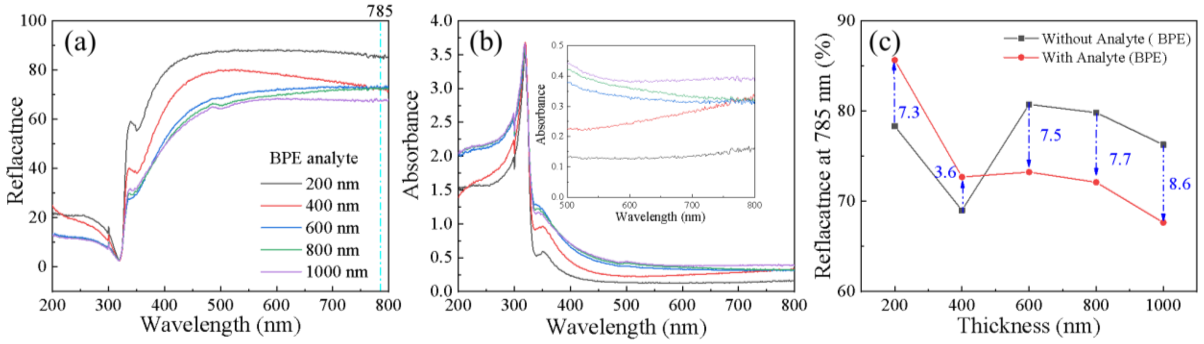

Figure S3. (a) Reflectance spectra of AgNR substrates after BPE adsorption. (b) Corresponding effective extinction spectra. (c) Comparison of reflectance and effective extinction at 785 nm for bare and BPE-coated substrates.

To evaluate the influence of molecular adsorption, reflectance and effective extinction were also measured after BPE adsorption (**Figure S3**). Although modest variations in magnitude are observed, including approximately 3–8% changes in reflectance at 785 nm depending on

nanorod length, the spectral response remains broadband and no distinct resonance feature is introduced. These results confirm that BPE adsorption does not significantly alter the collective plasmonic character of the AgNR-on-film architecture.

Overall, the optical measurements demonstrate that the substrates exhibit a collective broadband plasmonic response rather than a sharp LSPR peak aligned with the excitation wavelength.

S3. Additional Experimental Details: Defect Mapping

To assess how local substrate defects contribute to spectral variability, spatially resolved SERS mapping was performed on AgNR substrates coated with BPE. Mapping was conducted under identical acquisition conditions (fixed laser power, integration time, and step size) to isolate substrate-induced effects. This experiment was designed to identify spatial heterogeneity in electromagnetic enhancement arising from defects, nanorod non-uniformity, or mechanical damage, which is a known source of point-to-point intensity fluctuations in SERS measurements. By correlating SERS intensity variations with the physical substrate morphology, this analysis establishes that localized structural imperfections directly suppress Raman enhancement, leading to increased spectral variation.

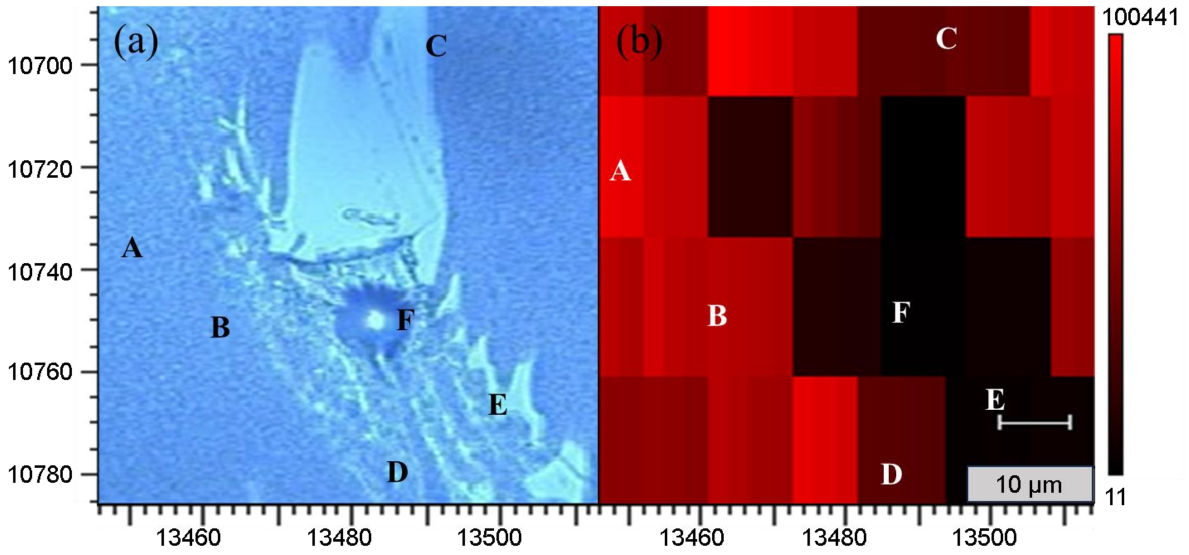

Figure S4. Spatially resolved defect mapping on AgNR substrates. (a) Optical micrograph of the mapped region, showing structural defects and the six selected locations (A–F). These positions represent distinct surface environments ranging from defect-free areas to regions directly on or near visible defects. (b) SERS intensity heat map of the same area, constructed from the 1204 cm^{-1} BPE Raman band. Brighter pixels correspond to stronger Raman enhancement, whereas darker pixels indicate low-signal regions.

S4. Additional Hierarchical Clustering Analysis: Selecting Subclusters within High-SNR and Low-SNR Groups

To determine the optimal number of clusters for the spectral datasets, both the Elbow method and silhouette analysis were applied independently to the high-SNR and low-SNR groups. The Elbow method evaluates the reduction in within-cluster variance as the number of clusters increases, while the silhouette score quantifies cluster separation by comparing intra-cluster compactness with inter-cluster distance. These results support the selection of seven clusters, providing an optimal trade-off between interpretability, intra-cluster homogeneity, and inter-cluster separation. This analysis justifies the use of the seven-label classification (Clusters I–VII) adopted throughout the main manuscript.

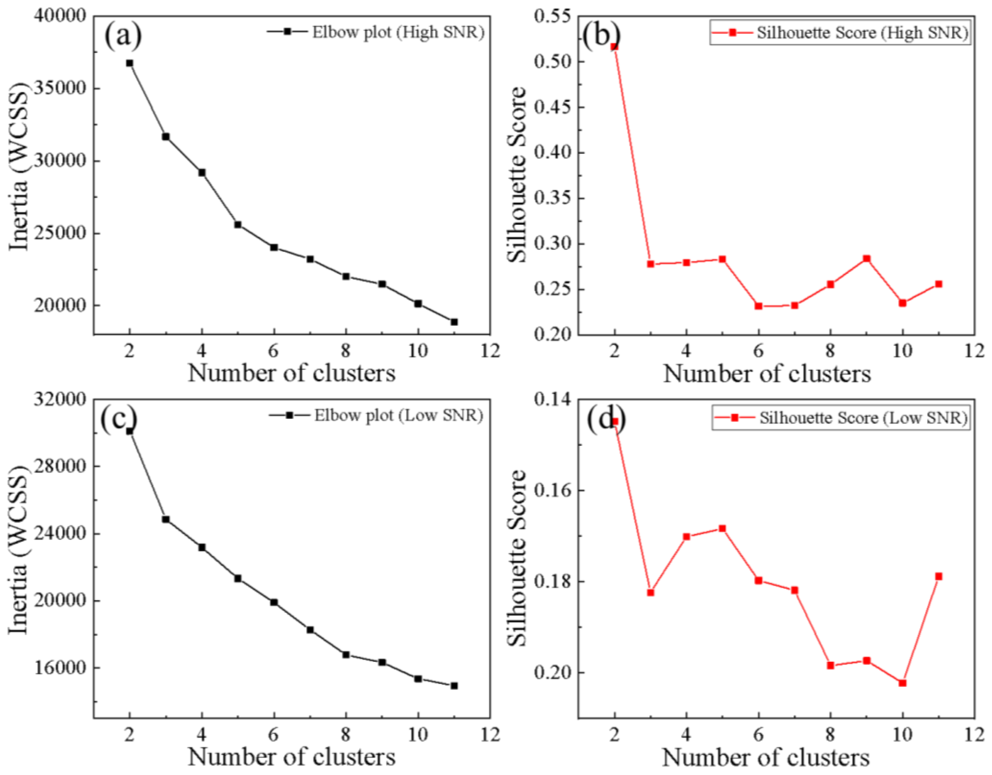

Figure S5. Determination of the number of subclusters within the High-SNR and Low-SNR spectral groups. (a) Elbow plot and (b) mean silhouette score versus cluster number for the High-SNR group. (c) and (d) silhouette analysis for the Low-SNR group, used to assess subcluster separation and guide selection of the final subcluster count

S5. Additional Information about Defect Mapping Metadata

This section provides supporting definitions for the metadata labels, including those associated with the defect-mapping experiments, referenced in the main text and used for the cluster–condition statistical analysis.

For defect-mapping experiments, spectra were classified based on their spatial relationship to visible substrate defects. Defect-on spectra include measurements acquired directly on the defect region as well as from positions immediately surrounding the defect. Defect-off spectra were collected from structurally uniform regions located far from the defect. The spatial boundary used for this classification is illustrated in **Figure S6a**, where the dashed circle indicates the approximate region used to distinguish between defect-on and defect-off measurements.

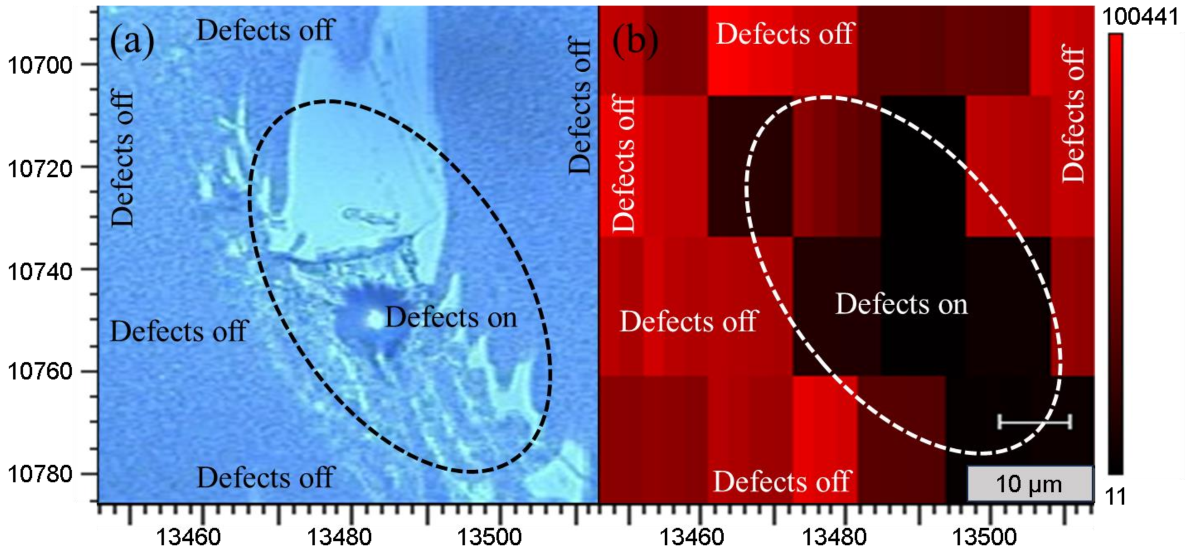

Figure S6. Defect on / near defect / defect off mapping regions on AgNR substrates. (a) Optical micrograph of the mapped area showing the spatial location of the structural defect (dashed circle), the surrounding "near defect" region, and the outer "defect off" region. (b) Corresponding SERS intensity map at 1204 cm^{-1} for the same lattice positions.

S6. Electromagnetic Weighting and Orientation-Dependent Raman Tensor Projections for BPE on AgNR

To rigorously clarify the role of electromagnetic enhancement and molecular orientation in the normalized SERS spectra, we present a complete tensor-based framework that explicitly incorporates the following components:

a) Electromagnetic (EM) field weighting
b) Surface selection rule
c) Coordinate transformations
d) Mode-specific polarizability anisotropy

This formalism explains the orientation-dependent sensitivities observed for the 1645, 1615, 1344, 1204, and 1015 cm^{-1} modes of BPE justifies the use 1015 cm^{-1} peak for normalization.

S6.1 General SERS intensity expression

The SERS intensity of the m-th vibrational mode can be expressed as

$$I_m \propto \left|\vec{E}_{loc}(\lambda_{ex})\right|^2 \left|\vec{E}_{loc}(\lambda_{sc,m})\right|^2 \left|\hat{e}_{sc}.\vec{\alpha}^{(m)}.\hat{e}_{local}\right|^2 \qquad (S1)$$

where $\vec{E}_{loc}(\lambda)$ is the local electric field at the excitation (λ_{ex}) or scattered $(\lambda_{sc,m})$ wavelength, $\vec{\alpha}^{(m)} \approx \left(\frac{\partial \vec{\alpha}}{\partial Q_v}\right)$ is the Raman polarizability tensor of mode m, $\hat{e}_{sc}$ and $\hat{e}_{local} = \frac{\vec{E}_{local}}{|\vec{E}_{local}|}$ are the scattered and local field directions. Thus, the SERS intensity consists of two independent contributions[4-7]:

- Electromagnetic (EM) enhancement weighting: $\left|\vec{E}_{loc}(\lambda_{ex})\right|^2 \left|\vec{E}_{loc}(\lambda_{sc,m})\right|^2$
- Raman tensor projection (orientation dependence): $\left|\hat{e}_{sc}.\vec{\alpha}^{(m)}.\hat{e}_{local}\right|^2$

S6.2 Local hotspot coordinate system and surface selection rule

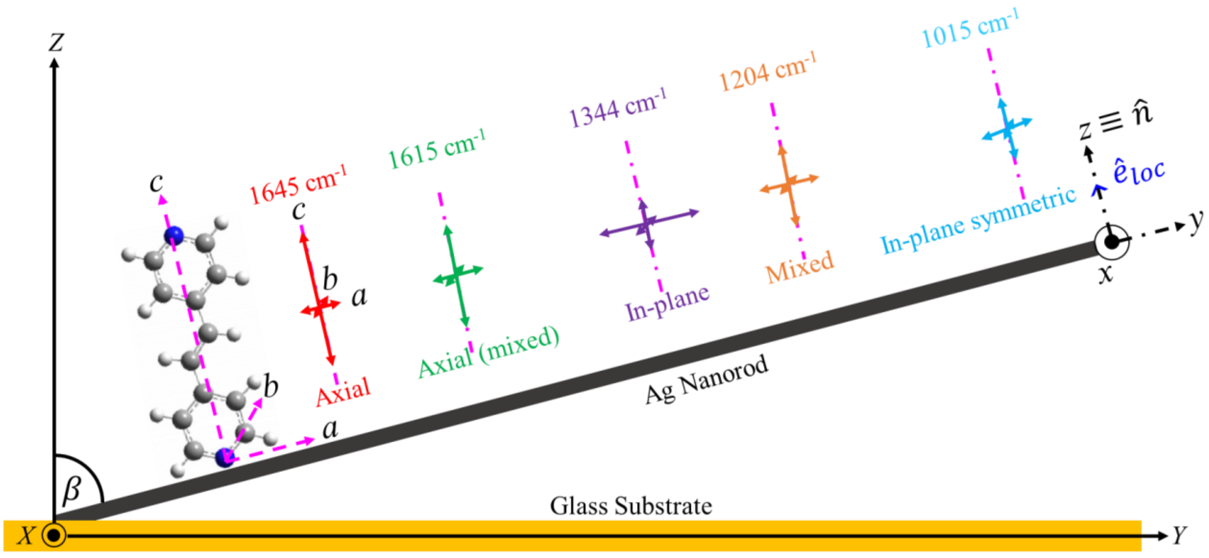

Figure S8. Schematic of upright adsorption geometry showing lab frame (X, Y, Z), local hotspot frame $(x, y, z$; $z \equiv$ surface normal), and molecular principal axes (a, b, c). The local enhanced electric field $\vec{E}_{local}$ is aligned with the surface normal.

Three coordinate systems are used as shown in **Figure S8**:

1. Global lab frame (X, Y, Z)
2. Local hotspot (nanorod) frame (x, y, z)
3. Molecular principal-axis frame (a, b, c)

We define a local hotspot frame (x, y, z) such that:

- $z \equiv \hat{n}$ local surface normal direction
- x, y tangential directions

For a BPE molecule adsorbed on the nanostructure shown in **Figure S8**, the molecular principal axes are defined as:

- c: molecular long axis
- a: in-plane short axis

- b: out-of-plane axis (normal to molecular plane)

For metallic nanostructures such as Ag nanorods, the enhanced near-field is dominated by the surface-normal component (surface selection rule)[8, 9]. We therefore approximate $\hat{e}_{local} \approx \hat{n}$. Under this approximation and for a vertically adsorbed BPE molecule on nanorod surface shown in **Figure S8**, **Eq. S1** can be simplified to,

$$I_m \propto |E_z(\lambda_{ex})|^2 |E_z(\lambda_{sc})|^2 \left|\vec{\alpha}_{zz}^{(m)}\right|^2. \qquad (S2)$$

Thus, orientation effects are governed by the projection of the Raman tensor onto the local z-axis. Although the Ag nanorods are globally tilted (~72–75°), this tilt is inherently incorporated by defining the local z-axis along the dominant near-field polarization direction. Since SERS intensity depends only on the projection of the Raman tensor onto the local near-field direction, incorporating the global nanorod tilt explicitly does not alter the relative mode-dependent tensor projections.

S6.3 Raman tensor in the molecular frame

In the molecular principal-axis system,

$$\vec{\alpha}_{mol}^{(m)} \approx \begin{pmatrix} \alpha_{aa}^{(m)} & 0 & 0 \\ 0 & \alpha_{bb}^{(m)} & 0 \\ 0 & 0 & \alpha_{cc}^{(m)} \end{pmatrix}. \qquad (S3)$$

The tensor in the nanorod frame is obtained via rotation,

$$\vec{\alpha}_{NR}^{(m)} = R\vec{\alpha}_{mol}^{(m)} R^T. \qquad (S4)$$

The measurable quantity is $\left(\vec{\alpha}_{NR}^{(m)}\right)_{zz}$.

S6.4 Mode-specific polarizability anisotropy of BPE

Based on reported vibrational analyses and DFT calculations of BPE on silver surfaces[10-12], **Table S1** summarizes the dominant Raman tensor components for the five modes discussed in the main text.

Table S1. Dominant Raman tensor anisotropy of the five characteristic BPE vibrational modes.

Peak (cm^{-1})	Mode	Tensor properties
1645	C=C stretch	$\alpha_{cc}^{(1645)} \gg \alpha_{aa}^{(1645)} \gg \alpha_{bb}^{(1645)}$
1615	Aromatic ring stretch	$\alpha_{cc}^{(1615)} > \alpha_{aa}^{(1615)} \gg \alpha_{bb}^{(1615)}$
1344	Ring deformation (tangential-dominated)	$\alpha_{aa}^{(1344)} > \alpha_{cc}^{(1344)} \gg \alpha_{bb}^{(1344)}$
1204	In-plane C–H bending and C–C stretching (mixed axial + tangential character)	$\alpha_{cc}^{(1204)} \gtrsim \alpha_{aa}^{(1204)} \gg \alpha_{bb}^{(1204)}$
1015	Ring breathing	$\alpha_{cc}^{(1015)} \sim \alpha_{aa}^{(1015)} \gg \alpha_{bb}^{(1015)}$

S6.5 Orientation-dependent tensor projections

To quantitatively evaluate how molecular orientation modifies the measurable tensor projection $\vec{\alpha}_{NR}$, we consider successive rotations of the molecular frame relative to the local hotspot frame. These rotations allow direct identification of mode-dependent orientation sensitivities. Below, we analyze distinct rotational geometries and their mode-dependent consequences.

Rotation about the *x*-Axis (in-plane tilt)

We first consider rotation of the molecule by an angle θ about the x-axis, corresponding to tilting in the *yz* plane, i.e., the plane of the tilted nanorods.

Under this transformation, the projection becomes,

$$\left(\vec{\alpha}_{NR}^{(m)}\right)_{zz} = \alpha_{aa}^{(m)} \sin^2\theta + \alpha_{cc}^{(m)} \cos^2\theta \,. \qquad (S5)$$

Only the in-plane tensor components $\alpha_{cc}^{(m)}$ and $\alpha_{aa}^{(m)}$ contribute to SERS intensity; the out-of-plane component $\alpha_{bb}^{(m)}$ does not enter this expression. Therefore, orientation sensitivity under this rotation is governed by the anisotropy

$$\Delta = \left|\alpha_{cc}^{(m)} - \alpha_{aa}^{(m)}\right|.$$

The limiting cases are

- $\theta_y = 0 \rightarrow \left(\vec{\alpha}_{NR}^{(m)}\right)_{zz} = \alpha_{cc}^{(m)}$
- $\theta_y = 90^0 \rightarrow \left(\vec{\alpha}_{NR}^{(m)}\right)_{zz} = \alpha_{aa}^{(m)}$

Thus, a large anisotropy $\left|\alpha_{cc}^{(m)} - \alpha_{aa}^{(m)}\right|$ leads to strong orientation sensitivity, whereas small anisotropy results in weak orientation dependence.

If $\alpha_{cc}^{(m)} \gg \alpha_{aa}^{(m)}$, then $\vec{\alpha}_{zz}^{m} \propto \alpha_{cc}^{(m)} \cos^2\theta \Rightarrow I_m \propto \cos^4\theta$, indicating strong intensity decrease with increasing tilt.

If $\alpha_{aa}^{(m)} \gg \alpha_{cc}^{(m)}$, then $\vec{\alpha}_{zz}^{(m)} \propto \alpha_{aa}^{(m)} \sin^2\theta \Rightarrow I_m \propto \sin^4\theta$, indicating intensity increases with tilt

If $\alpha_{cc}^{(m)} \approx \alpha_{aa}^{(m)}$, then $\left(\vec{\alpha}_{NR}^{(m)}\right)_{zz} = \alpha_{aa}^{(m)} \sin^2\theta + \alpha_{cc}^{(m)} \cos^2\theta \approx \alpha^{(m)}{}_{iso}$, intensity becomes nearly orientation-independent.

Based on these relations, the expected in-plane tilt sensitivity for each BPE vibrational mode is summarized in **Table S2**.

Table S2. Expected tilt sensitivity of BPE vibrational modes under rotation about the x-axis (tilt in the *y-z* plane)

Peak (cm^{-1})	In-Plane Tensor Relation	In plane anisotropy $\left\|\alpha_{cc}^{(m)} - \alpha_{aa}^{(m)}\right\|$
1645	$\alpha_{cc}^{(1645)} \gg \alpha_{aa}^{(1645)}$	Very Strong
1615	$\alpha_{cc}^{(1615)} > \alpha_{aa}^{(1615)}$	Strong

1344	$\alpha_{aa}^{(1344)} > \alpha_{cc}^{(1344)}$	Strong
1204	$\alpha_{cc}^{(1204)} \gtrsim \alpha_{aa}^{(1204)}$	Moderate
1015	$\alpha_{cc}^{(1015)} \sim \alpha_{aa}^{(1015)}$	Nearly invariant

Therefore, under the surface selection rule and upright adsorption geometry of BPE on AgNR, tilt about the x-axis directly modulates the relative projection of $\alpha_{cc}^{(m)}$ and $\alpha_{aa}^{(m)}$ and constitutes the dominant orientation-dependent mechanism in the present system.

Rotation about the y-axis would correspond to bending the molecule toward or away from the surface. Because BPE binds through the pyridyl nitrogen and favors an upright adsorption geometry, large bending motions are energetically unfavorable and do not significantly modify the surface-normal tensor projection.

Azimuthal rotation about the surface normal corresponds to in-plane twisting around the anchoring site. Due to distributed azimuthal adsorption across the AgNR surface, ensemble averaging effectively restores rotational symmetry about the surface normal. Consequently, azimuthal rotation does not produce systematic mode-dependent intensity variation.

Therefore, based on the results in **Table S2**, and the statistic argument regarding molecule azimuthal distribution, the 1015 cm^{-1} ring breathing mode intensity is insensitive to orientation change compared to other modes. Thus, the normalization using the $I^{(1015)}$ can reflect the orientation changes of other modes.

Reference

(1) Zhao, Y.; Kumar, A.; Yang, Y. Unveiling practical considerations for reliable and standardized SERS measurements: lessons from a comprehensive review of oblique angle deposition-fabricated silver nanorod array substrates. *Chem. Soc. Rev.* **2024**, *53* (2), 1004-1057.
(2) Gabudean, A.; Biro, D.; Astilean, S. Localized surface plasmon resonance (LSPR) and surface-enhanced Raman scattering (SERS) studies of 4-aminothiophenol adsorption on gold nanorods. *J. Mol. Struct.* **2011**, *993* (1–3), 420–424.
(3) Willets, K. A.; Van Duyne, R. P. Localized surface plasmon resonance spectroscopy and sensing. *Annu. Rev. Phys. Chem.* **2007**, *58* (1), 267-297.
(4) Moskovits, M. Surface-enhanced spectroscopy. *Rev. Mod. Phys.* **1985**, *57* (3), 783.
(5) Le Ru, E.; Etchegoin, P. *Principles of Surface-Enhanced Raman Spectroscopy: and Related Plasmonic Effects*; Elsevier, 2008.
(6) Yamamoto, Y. S.; Itoh, T. Why and how do the shapes of surface-enhanced Raman scattering spectra change? Recent progress from mechanistic studies. *J. Raman Spectrosc.* **2016**, *47* (1), 78-88.
(7) Yoshida, K.-i.; Itoh, T.; Tamaru, H.; Biju, V.; Ishikawa, M.; Ozaki, Y. Quantitative evaluation of electromagnetic enhancement in surface-enhanced resonance Raman scattering from plasmonic properties and morphologies of individual Ag nanostructures. *Phys. Rev. B* **2010**, *81* (11), 115406.
(8) Moskovits, M. Surface selection rules. *J. Chem. Phys.* **1982**, *77* (9), 4408-4416.
(9) Long, D. A. *The Raman Effect*; John Wiley & Sons, 2002.
(10) Yang, W. h.; Hulteen, J.; Schatz, G. C.; Van Duyne, R. P. A surface-enhanced hyper-Raman and surface-enhanced Raman scattering study of trans-1, 2-bis (4-pyridyl) ethylene adsorbed onto silver film over nanosphere electrodes. Vibrational assignments: Experiment and theory. *J. Chem. Phys.* **1996**, *104* (11), 4313-4323.

(11) Zhuang, Z.; Cheng, J.; Jia, H.; Zeng, J.; Han, X.; Zhao, B.; Zhang, H.; Zhang, G.; Zhao, W. Density functional theory calculation of vibrational spectroscopy of trans-1, 2-bis (4-pyridyl)-ethylene. *Vib. Spectrosc.* **2007**, *43* (2), 306-312.
(12) Zhuang, Z.; Shi, X.; Chen, Y.; Zuo, M. Surface-enhanced Raman scattering of trans-1, 2-bis (4-pyridyl)-ethylene on silver by theory calculations. *Spectrochim. Acta, Part A* **2011**, *79* (5), 1593-1599.
